# Identification of mechanosensitive genes during skeletal development: alteration of genes associated with cytoskeletal rearrangement and cell signalling pathways

**DOI:** 10.1186/1471-2164-15-48

**Published:** 2014-01-20

**Authors:** Rebecca A Rolfe, Niamh C Nowlan, Elaine M Kenny, Paul Cormican, Derek W Morris, Patrick J Prendergast, Daniel Kelly, Paula Murphy

**Affiliations:** 1 Department of Zoology, School of Natural Sciences, Trinity College Dublin, Dublin, Ireland; 2 Trinity Centre for Bioengineering, School of Engineering, Trinity College Dublin, Dublin, Ireland; 3 TrinSeq, Institute of Molecular Medicine and Department of Psychiatry, Trinity College Dublin, Dublin, Ireland; 4 Department of Bioengineering, Imperial College London, London, UK

**Keywords:** Skeletal development, Mechanical stimulation, Transcriptome, Mechanosensitive genes, Wnt signalling, Spd, Cytoskeleton

## Abstract

**Background:**

Mechanical stimulation is necessary for regulating correct formation of the skeleton. Here we test the hypothesis that mechanical stimulation of the embryonic skeletal system impacts expression levels of genes implicated in developmentally important signalling pathways in a genome wide approach. We use a mutant mouse model with altered mechanical stimulation due to the absence of limb skeletal muscle (*Splotch-delayed*) where muscle-less embryos show specific defects in skeletal elements including delayed ossification, changes in the size and shape of cartilage rudiments and joint fusion. We used Microarray and RNA sequencing analysis tools to identify differentially expressed genes between muscle-less and control embryonic (TS23) humerus tissue.

**Results:**

We found that 680 independent genes were down-regulated and 452 genes up-regulated in humeri from muscle-less Spd embryos compared to littermate controls (at least 2-fold; corrected p-value ≤0.05). We analysed the resulting differentially expressed gene sets using Gene Ontology annotations to identify significant enrichment of genes associated with particular biological processes, showing that removal of mechanical stimuli from muscle contractions affected genes associated with development and differentiation, cytoskeletal architecture and cell signalling. Among cell signalling pathways, the most strongly disturbed was Wnt signalling, with 34 genes including 19 pathway target genes affected. Spatial gene expression analysis showed that both a Wnt ligand encoding gene (*Wnt4*) and a pathway antagonist (*Sfrp2*) are up-regulated specifically in the developing joint line, while the expression of a Wnt target gene, *Cd44*, is no longer detectable in muscle-less embryos. The identification of 84 genes associated with the cytoskeleton that are down-regulated in the absence of muscle indicates a number of candidate genes that are both mechanoresponsive and potentially involved in mechanotransduction, converting a mechanical stimulus into a transcriptional response.

**Conclusions:**

This work identifies key developmental regulatory genes impacted by altered mechanical stimulation, sheds light on the molecular mechanisms that interpret mechanical stimulation during skeletal development and provides valuable resources for further investigation of the mechanistic basis of mechanoregulation. In particular it highlights the Wnt signalling pathway as a potential point of integration of mechanical and molecular signalling and cytoskeletal components as mediators of the response.

## Background

Mechanical stimulation plays an important role in skeletal growth and repair reviewed in [1] and, although much less well studied, it is also required for normal skeletal development. This was initially indicated by observations that infants who experience decreased foetal movement *in utero* due to neuromuscular disorders present a range of skeletal anomalies including multiple joint fusions, craniofacial abnormalities and thin hypo-mineralised bones [2,3]. Direct evidence that mechanical stimuli generated by embryonic muscle contractions impacts skeletal development comes from a variety of experimental animal models that show similar abnormalities in ossification and joint formation, for example following muscle immobilisation in chick embryos, and in mouse embryos lacking muscle or with reduced or immobile muscle reviewed in [4,5]. However little is known about the molecular mechanisms through which mechanical stimuli influence cellular events during skeletal development. The interplay between biophysical stimuli and gene regulation in differentiating cells is emerging as an important phenomenon in multiple developmental systems [6,7].

A number of different strains of mutant mice have been studied that phenotypically lack limb muscle or show reduced stimuli from muscle contraction during development [8-10], including *Splotch* (Pax3^Sp^) and *Splotch-delayed* (Pax3^Spd^), where muscle precursor cells fail to migrate to the developing limbs and no limb muscle forms [11,12]. Common defects in muscle-less and immobilised embryos include abnormal initiation and/or progression of ossification [9,13], loss of definition of tissue territories in the joint region [8] and altered rudiment morphology [9], associated with reduced local cell proliferation [14]. Therefore, mechanical stimuli impact a variety of developmental processes and presumably must influence or integrate with signalling pathways and molecular changes known to guide these events. One clue to a signalling pathway impacted by mechanical stimulation comes from the work of Kahn *et al.*[8] who showed that canonical Wnt signalling is altered in the elbow joint of *Splotch-delayed* embryos. Several regulatory genes have been shown to have dramatically altered expression patterns in reduced mechanical stimuli including, *Ihh* and *ColX* at the site of ossification [10,15] and *Bmp2*, *Fgf2*, and *Pthlp* at the joint line [16,17]. Whether expression of these genes is directly affected by the mechanical environment or as a more indirect consequence of altered cell behaviour is not known; a genome-wide, open ended screen is required to know more about the spectrum of molecular changes that occur when mechanical stimuli are altered.

Gene expression profiling to identify genome-wide changes under altered mechanical environments has been carried out on cells in culture using microarray technology, including osteoblast cell-lines subjected to weightlessness or microgravity conditions [18], chondrocyte-laden constructs and murine cartilage explants to which dynamic compression was applied [19] and chondrocyte cell lines exposed to hydrostatic pressure [20]. Gene expression profiling has the potential to uncover hundreds of genes that respond to mechanical stimuli simultaneously (mechanosensitive genes); however no direct analyses of *in vivo* changes in gene expression during skeletal development following alteration of the mechanical environment have been performed. This is required to begin to assemble a picture of the molecular landscape impacted by mechanical stimuli in a developmental context.

In this study we analysed the transcriptional changes in the developing humerus and associated joints at Theiler stage (TS) 23 [21] (typically embryonic day (E) 14.5) in muscle-less (*Splotch-delayed*) compared to phenotypically normal littermate controls. We previously established that the humerus is the most strongly affected rudiment and TS23 the earliest time point at which the specific effects on ossification and joint line reduction in the elbow and shoulder regions are detected [9,10]. We hypothesise that mechanical stimulation of the embryonic skeletal system impacts expression levels of genes implicated in a variety of regulatory pathways and biological processes, as would be expected when an integrated regulatory system is disturbed. The genes that show altered expression would include direct and indirect targets of mechanical stimulation. Therefore, a genome wide analysis of altered transcript levels is required to indicate the principal molecular mechanisms disturbed and the most likely candidates for direct regulation. We have used both RNA whole transcriptome sequencing analysis (RNA-seq) and Microarray technology to allow a comprehensive investigation of the altered transcriptome. Microarray analysis is a more established technique [22], but RNA-seq offers the potential of greater sensitivity [23] and analysing the same tissues in parallel allows direct comparison of the two assays and integration of the data sets. We also used RNA-seq analysis of the normal developing humerus to explore the transcriptome at this specific stage of development. The humerus developing in the absence of muscle generated stimulation showed both up and down-regulation of gene expression. We reveal alteration of genes encoding components and targets of specific signalling pathways, in particular the Wnt signalling pathway. Genes associated with cytoskeletal rearrangement and extracellular matrix components are also affected. This analysis has allowed us to profile genome wide transcriptional changes giving an overview of the molecular processes and pathways most affected and identifying a set of putative direct target genes responding to mechanical stimulation during ossification and joint formation.

## Results

### Transcription profiling of the developing humerus during early ossification and joint formation (TS23)

**Figure 1:**
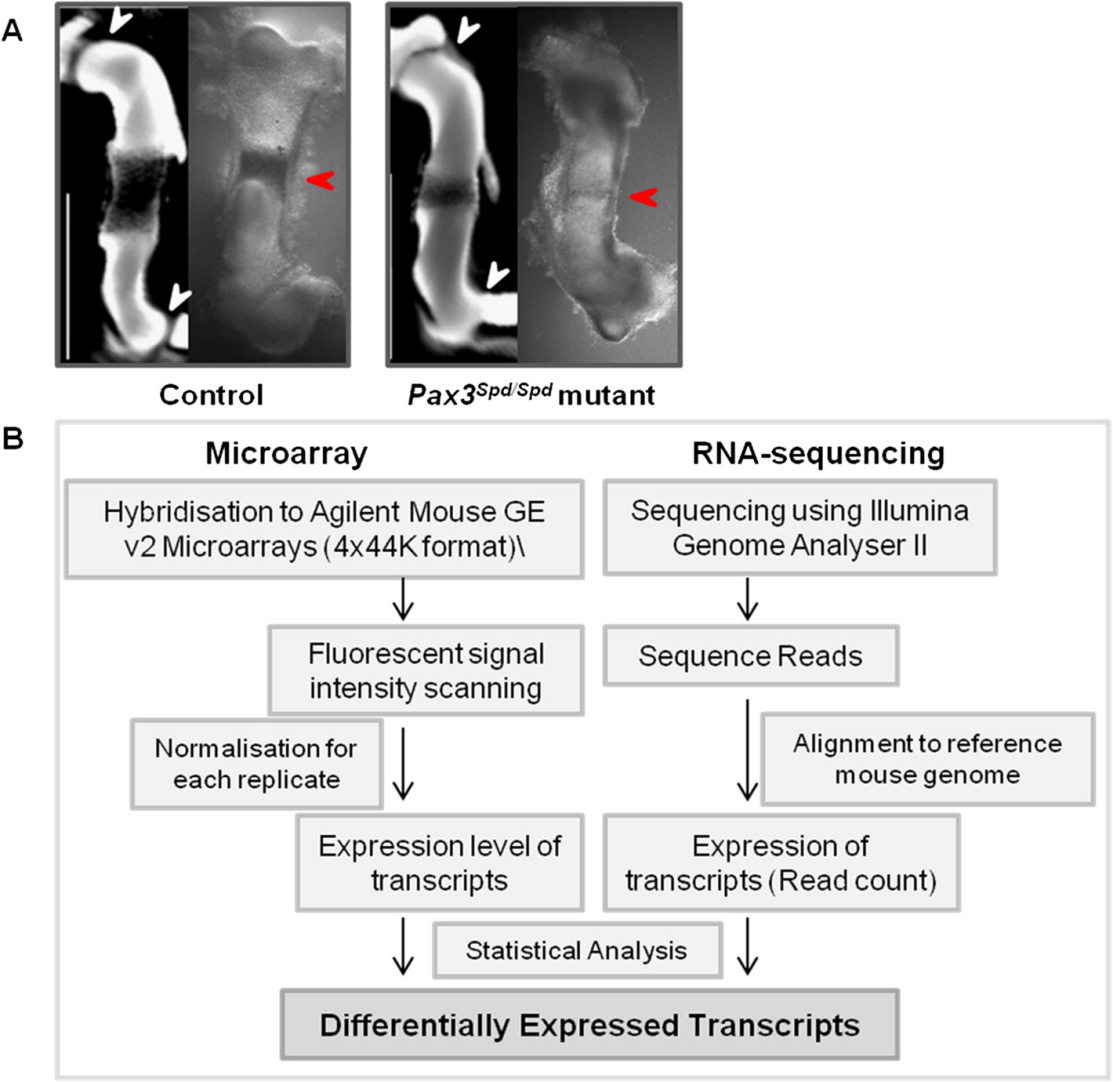
**Overview of gene expression profiling approach used. A)** Images of control and *Pax3*^*Spd/Spd*^ mutant humeri at Theiler stage 23 dissected prior to RNA extraction (right hand image); the images on the left are external views of stage and genotype matched 3D scanned specimens stained to reveal the morphology and ossification more clearly. White arrow heads indicate the elbow joint and shoulder joint line and red arrow heads the ossification site, a visible reduction is apparent in the mutant compared to the control in each case. **B)** Work-flow of Microarray and RNA-sequencing data analysis.

**Figure 2:**
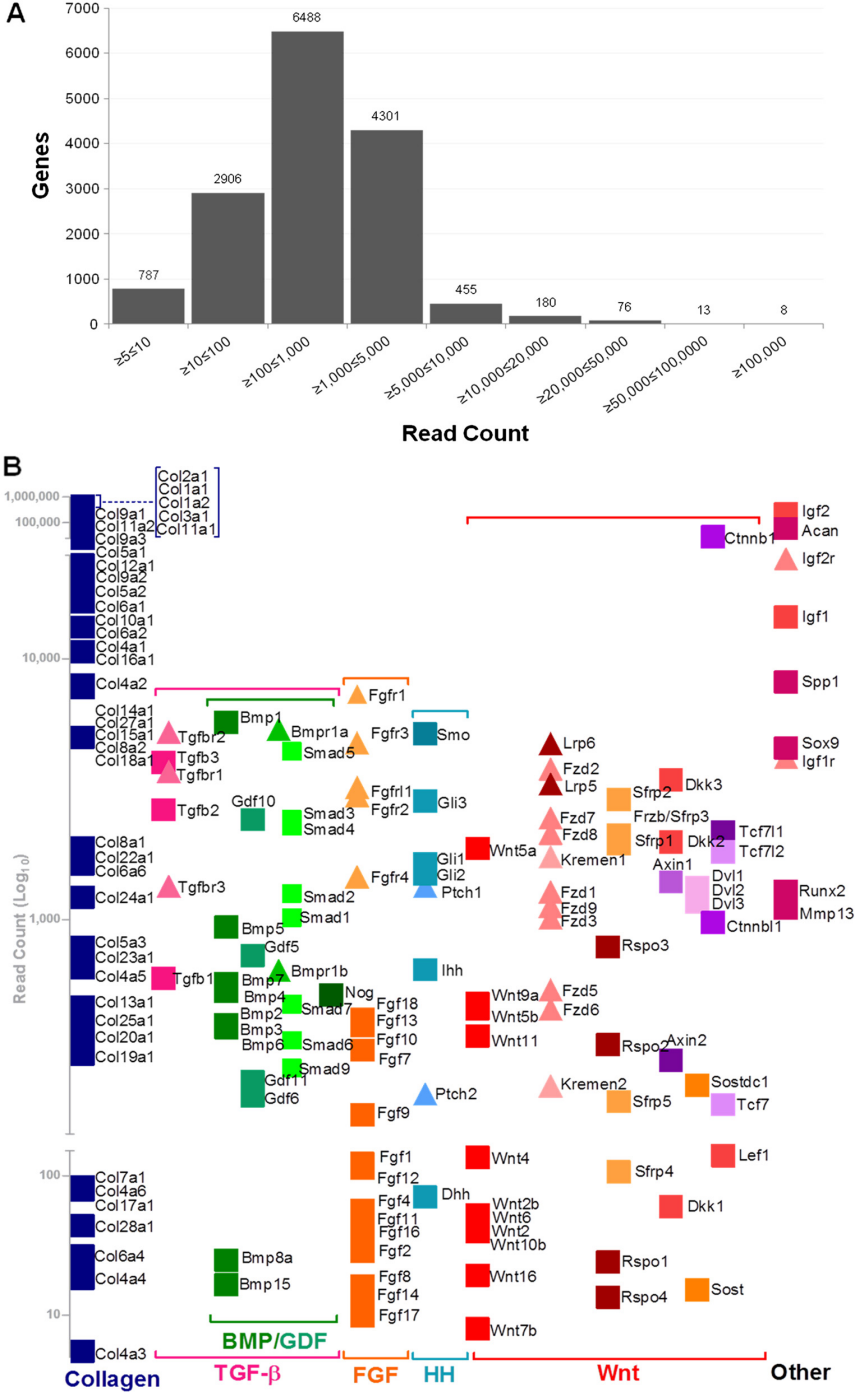
**Transcriptome Profiling of the developing humerus at TS23 by RNA-seq, (A) The number of genes (y-axis) with increasing relative expression levels represented by transcript read counts as indicated. (B)** The relative expression of selected genes represented by read counts (Log_10_) (y-axis). Collagen genes and signalling pathways genes are grouped: TGFβ (including TGFβ, BMP and GDF ligands, receptors and agonists), FGF (ligands and receptors), HH (ligands, receptors and intracellular modulators), Wnt (Wnt ligands and agonists (Wnt, Rspo), receptors (Fzd) and co-receptors (Kremen, Lrp), antagonists (Sfrp, Dkk) Intracellular components (Sost, Dvl, Ctnnb) and nuclear components (Tcf, Lef)) and a selection of ‘other’ genes associated with skeletal development. Squares indicate ligands and modulators, triangles indicate receptors.

RNA sequencing of control humeri at TS23 gives an insight into the transcriptome at this key stage of development when the rudiment is undergoing early stages of ossification and tissue zones in the joint are being defined (Figure 1). Transcripts from 15,214 individual genes (n = 3 biological replicates, Figure 2; data submitted to EMBL-EBI ArrayExpress repository: [E-MTAB-1745]) were detected in this tissue at this stage. A minimum of 5 transcript reads (on average), with at least one read from each replicate sample, was chosen as the cut off point to reliably indicate reproducible expression across biological replicates [24]. Expressed genes were divided into groups according to their relative expression level (Figure 2A). 787 genes showed the lowest level of expression represented by between 5 and 10 read counts, while the majority of genes (71%; 10,789/15,214) showed between 100 and 5000 read counts. Only 732 genes are in the most highly expressed categories (≥5,000 read counts) (Figure 2A), representing 4.8% of expressed genes. Expression levels of selected individual genes are represented in Figure 2B. The most abundantly transcribed gene is *Col2a1* (collagen 2, alpha 1) with a read count of 452,576, and among the 8 genes with read counts of more than 100,000, there are 4 other collagen encoding genes (Figure 2A and B). In total 41 collagen subtype genes are expressed (Figure 2B).

The relative levels of expression of genes associated with signalling pathways involved in regulating skeletal development reviewed in [25,26] are highlighted in Figure 2B. This shows the potential components that can contribute to these signalling pathways at this stage of skeletal development. For example the hedgehog (HH) pathway is known to play an important role in ossification through the action of Ihh (640 reads), binding to its receptor Ptch1 (1,342 reads) activating Smo (5,154 reads). Similarly, 14 FGF ligand encoding genes and 5 FGF receptor encoding genes were detected, highlighting the potential for multiple FGF signalling interactions. The BMP signalling pathway genes also reveal potential for multiple signalling interactions with 9 BMP encoding genes expressed. *Bmp1* is by far the most highly expressed (5719 reads); although it is not previously reported in this tissue at this stage in gene expression databases (http://www.informatics.jax.org/). The relative expression levels of Wnt ligand (*Wnt*), Wnt receptor (*Fzd*, *Lrp, Kremen*), extracellular Wnt interactor (*Sfrp*, *Dkk*, *Rspo, Sost*), intracellular Wnt pathway component (*Dvl*, *Axin*, *Ctnnb*) and Wnt pathway transcription factor (*Tcf*, *Lef*) encoding genes are represented. The detection of previously unreported Wnt gene expression in the humerus (e.g. *Wnt2* and *Wnt2b*) opens up new considerations for functional roles, especially as both genes are up-regulated in muscle-less rudiments (described below). The low density lipoprotein receptor-related genes *Lrp5* and *Lrp6*, which are Wnt co-receptors, are most highly expressed among the Lrp gene family (4,727 and 3,310 reads respectively). Interestingly eight Fzd receptor encoding genes are detected. Other genes known to be involved in skeletal development are highly expressed; *Sox9* (4,550 reads), *Runx2* (1,285reads), *Spp1* (8,155 reads) and *Mmp13* (1,103 reads).

### Identification of differentially expressed genes in muscle-less versus control developing humeri and associated joints

Microarray analysis of RNA extracted from control and *Pax3*^*Spd/Spd*^ muscle-less humeri (n = 4 biological replicates) detected expression of a similar proportion of individual genes on the array; 20,697 independent genes from the control and 20,949 from the muscle-less humeri (data submitted to EMBL-EBI ArrayExpress repository: E-MTAB-1744). Comparing hybridisation intensity between control and mutant derived cDNAs, using cut off points of at least a 2-fold change and corrected p-value ≤0.05 [27] for significance across replicates, identified 374 independent genes as differentially expressed (DE). Of these, 284 genes (75.9%) were down-regulated and 90 genes (24.1%) were up-regulated (Table 1). RNA-seq analysis (n = 3 biological replicates) detected 15,031 independent genes (with ≥5 read counts across replicates, as described above) in muscle-less humeri, compared to 15,214 in control tissue (data submitted to EMBL-EBI ArrayExpress repository: E-MTAB-1746). To determine differential expression, the same cut off points of a corrected p-value ≤0.05 and at least a 2-fold change were applied to the RNA-seq data-set, identifying 1,037 genes as DE across replicates. Of these, 618 genes (59.6%) were down-regulated and 419 genes (40.4%) were up-regulated in the muscle-less humeri and associated joints compared to that of phenotypically normal littermate control humeri (Table 1).

**Table 1:** Differentially expressed genes (p ≤0.05) with fold change values ≥ 2 revealed by Microarray and RNA-Seq analysis and Combined

	**Microarray**		**RNA-Seq**		**Combined**	
Total number of differentially expressed transcripts	374		1,037		1,132	
	**Down-regulated**	**Up-regulated**	**Down-regulated**	**Up-regulated**	**Down-regulated**	**Up-regulated**
Independent named genes	282	87	419	618	680	452
Putative uncharacterized proteins/unknown genes	2	3	-	-	-	-
	284	90	419	618		

**Figure 3:**
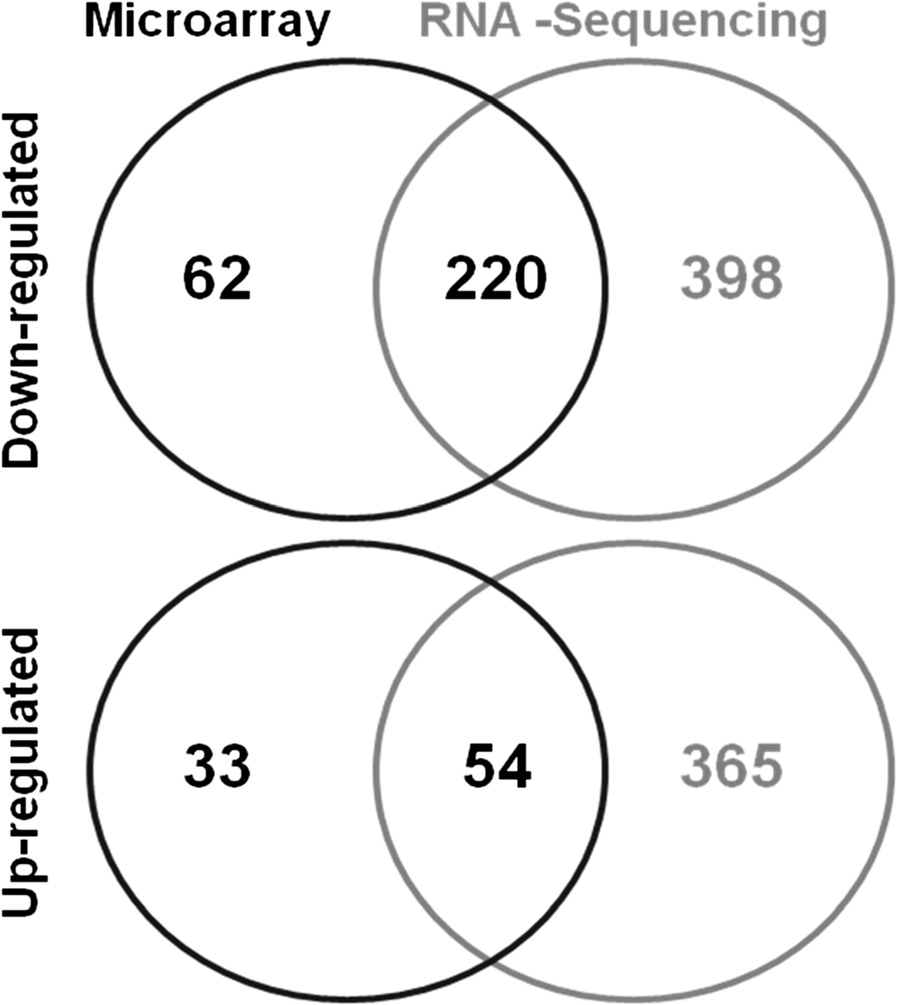
Venn diagrams representing the overlap of independent genes detected as differentially expressed (up-regulated and down-regulated; cut off criteria p ≤ 0.05, and fold change ≥2) from the microarray and RNA seq analysis, as indicated.

The Venn diagrams in Figure 3 represent overlap of DE genes identified by the two platforms. In total 1,132 independent genes were identified as DE in the muscle-less mutant compared to its phenotypic control (Figure 3, Table 1). Of the 374 genes identified by microarray (Table 1), 73.2% of these genes were also represented in the RNA-seq data. RNA-seq detected a greater total number of DE genes than the microarray (1,037 versus 374, respectively, using the standard cut off criteria). In addition if the stringency for DE gene selection is weakened for the microarray data by moving the cut off point to corrected p-value of ≤0.08, the number of genes detected as DE in common by the two platforms increases to 426 from 274 (not shown). This suggests that the RNA-seq approach was more sensitive in detecting differential expression. Grouping genes based on the degree of change showed that the spread of fold change values was similar across platforms although there were differences in the number of genes in different categories (Additional file 1: Table S1). Additional file 1: Table S2 and S3 list all DE genes and fold change values estimated by Microarray and RNA-seq.

Verification of fold change values for DE genes was performed using qRT-PCR (Table 2). The DE genes chosen (*Fgf4*, *Cilp*, *Rxrg*, *Dll1*, *Spp1*, *Vstm2a*, *Figf*, *Fgf10* and *Sfrp2)* include both down-regulated and up-regulated genes. The direction and degree of fold changes were similar in all cases for the microarray and the RNA-seq. For all genes analysed there was a good correspondence across all platforms (Microarray, RNA-seq and RT-PCR), although greater fold change differences were detected for the down-regulated gene *Rxrg* and the up-regulated gene *Vstm2a* by qRT-PCR.

**Table 2:** **Fold change in expression level of selected genes in *Pax3***^***Spd***^ ***/***^***Spd ***^**mutant humeri compared to control humeri revealed by Microarray, RNA seq and qRT-PCR**

**Gene symbol**	**Gene name**	**Regulation status**	**Microarray (Fold change)**	**RNA-seq (Fold change)**	**qRT-PCR (Fold change)**
*Fgf4*	Fibroblast growth factor 4	Down-regulated	41.05	#No numerical value	35.24^***^
*Cilp*	Cartilage intermediate layer protein, nucleotide pyrophosphohydrolase	Down-regulated	9.73	9.76	11.3^***^
*Rxrg*	Retinoid X receptor gamma	Down-regulated	7.05	12.29	5.25^**^
*Dll1*	Delta-like 1	Down-regulated	2.21	3.16	1.78^*^
*Spp1*	Secreted phospoprotein 1	Down-regulated	5.23^(ns)^	2.9	2.59^*^
*Vstm2a*	V-set and transmembrane domain containing 2A	Up-regulated	8.56	3.09	14.17^**^
*Figf*	c-fos induced growth factor	Up-regulated	3.52	3.17	4.06^**^
*Fgf10*	Fibroblast growth factor 10	Up-regulated	2.48	2.14	3.28
*Sfrp2*	Secreted frizzled-related protein 2	Up-regulated	2.62	2.09	3.38^*^

#No numerical value for fold change as no transcripts detected in the mutant.

***p ≤ 0.001, **p ≤ 0.01, *p ≤ 0.05 indicates significance of differential expression from qRT-PCR.

p ≤ 0.05 for all microarray and RNA-seq differences except *Spp1* in the Microarray (ns).

### Biological Interpretation of differentially expressed genes: Down-regulated genes are associated with development and differentiation, cytoskeletal architecture and cell signalling

To reveal any enrichment of functionally related genes among the DE data sets, two web based tools, DAVID and GOstat, were used to analyse Gene Ontology (GO) term associations [28,29] (Tables 3 and 4). A significant enrichment (normalised by the number of genes in the genome with that associated GO term) indicates specific biological processes that are affected when mechanical stimuli are reduced. The strength of the enrichment is indicated by the calculated p-value. Independent analysis of data sets from microarray and RNA-seq showed the same enriched groups so analysis of the combined DE gene sets is presented (Table 1). The individual GO terms found to be enriched have been grouped for the purpose of interpretation as indicated in Tables 3 and 4. Analysis of the down-regulated gene set (Table 3) indicated that genes associated with Development and Differentiation are most highly enriched (total of 155/680 with p-values down to 2.73 × 10^-15^). Categories within this group are involved in signal transduction, including genes that encode signalling molecules, receptors, and transcription factors (TFs), for example signalling ligands *Fgf4,Fgf5, Fgf6, Fgf8* from the fibroblast growth factor signalling pathway; receptors *Fzd10* and *Rxrg* from the Wnt and Retinoic acid pathways respectively. 26 of these genes encode TFs including; *Barx2*, *Scx, Hes6, Pitx2*, *Pitx3 and Tead4*. The down-regulation of such signalling pathway component genes also underlies the enrichment of ontology groups related to Cell Signalling (Table 3: p-value down to 4.78 × 10^-5^).

**Table 3:** Enrichment of gene groups down-regulated in muscle-less humeri based on GO terms under subontologies biological process and cellular component

**Gene ontology term**		**Gene count in study**	**Enrichment**^**1**^
**Development/Differentiation**		155	2.73 × 10^-15^ to 0.0084
	GO:0048856: Anatomical structure development	134	2.73 × 10^-15^
	GO:0032502: Developmental process	155	1.52 × 10^-14^
	GO:0048513: Organ development	107	4.23 × 10^-13^
	GO:0007275: Multicellular organismal development	142	4.92 × 10^-13^
	GO:0048731: System development	122	5.57 × 10^-13^
	GO:0009888: Tissue development	58	8.37 × 10^-13^
	GO:0030154: Cell differentiation	94	8.86 × 10^-10^
	GO:0048869: Cellular developmental process	96	1.67 × 10^-9^
	GO:0048468: Cell development	45	2.66 × 10^-7^
	GO:0009887: Organ morphogenesis	38	6.12 × 10^-5^
	GO:0001756: Somitogenesis	8	2.61 × 10^-4^
	GO:0035282: Segmentation	9	2.66 × 10^-4^
	GO:0009952: Anterior/posterior pattern formation	13	0.0033
	GO:0009790: Embryonic development	34	0.0074
	GO:0043009: Chordate embryonic development	24	0.0075
	GO:0009792: Embryonic development ending in birth	24	0.0084
**Cytoskeleton**		30	5.30 × 10^-19^ to 0.0995
Biological process	GO:0030029: Actin filament-based process	25	1.45 × 10^-9^
	GO:0030036: Actin cytoskeleton organization	23	1.08 × 10^-8^
	GO:0007010: Cytoskeleton organization	31	1.50 × 10^-7^
	GO:0007015: Actin filament organization	5	0.0995
	GO:0015629: Actin cytoskeleton	39	5.30 × 10^-19^
Cellular component	GO:0005856: Cytoskeleton	86	2.20 × 10^-14^
	GO:0016459: Myosin complex	21	1.26 × 10^-15^
	GO:0032982: Myosin filament	11	2.32 × 10^-12^
	GO:0044430: Cytoskeletal part	51	1.16 × 10^-6^
	GO:0005884: Actin filament	5	0.0134
**Cell signalling**		31	4.78 × 10^-5^ to 0.0899
	GO:0007154: Cell communication	32	4.78 × 10^-5^
	GO:0007267: Cell-cell signalling	23	1.33 × 10^-4^
	GO:0007169: Transmembrane protein tyrosine kinase signalling	17	3.85 × 10^-4^
	GO:0007167: Enzyme linked receptor protein signalling pathway	20	0.0011
	GO:0008543: Fibroblast growth factor receptor signalling pathway	5	0.0139
	GO:0045168: Cell-cell signalling involved in cell fate specification	4	0.0271
	GO:0040036: Regulation of fibroblast growth factor signalling	3	0.0451
	GO:0043409: Negative regulation of MAPKKK cascade	3	0.0614
	GO:0007219: Notch signalling pathway	5	0.0899

All GO terms are associated with the subontology “Biological Process” except where indicated under Cytoskeleton, where Cellular Component was also used.

^1^P-value of enrichment of GO terms using DAVID analysis software; similar results were found with GOstat.

**Table 4:** Enrichment of gene groups up-regulated in muscle-less humeri based on GO terms under subontologies biological process and cellular component

**Gene ontology: biological process**		**Gene count in study**	**Enrichment**^**1**^
**Adhesion/Extracellular matrix**		32	1.99 × 10^-10^ to 0.0842
GO:0007155: Cell adhesion		41	1.99E-10
GO:0022610: Biological adhesion		41	2.12E-10
GO:0007156: Homophilic cell adhesion		15	4.95 × 10^-7^
GO:0016337: Cell-cell adhesion		19	9.38 × 10^-6^
GO:0043062: Extracellular structure organization		9	0.0223
GO:0030198: Extracellular matrix organisation		6	0.0842
**Cell signalling**		33	3.45 × 10^-9^ to 0.0148
GO:0007267: Cell-cell signalling		27	3.45 × 10^-9^
GO:0007154: Cell communication		33	2.05 × 10^-9^
GO:0007223: Wnt receptor signalling pathway, calcium modulating pathway		7	0.0092
GO:0016055: Wnt receptor signalling pathway		4	0.0104
GO:0008589: Regulation of smoothened signalling pathway		9	0.0105
GO:0010648: Negative regulation of smoothened signalling pathway		4	0.0135
GO:0048010: Vascular endothelial growth factor receptor signalling pathway		10	0.0288
GO:0007169: Transmembrane receptor protein tyrosine kinase signalling pathway		3	0.0402
GO:0007167: Enzyme linked receptor protein signalling pathway		12	0.0051
GO:0007165: Signal transduction		80	0.0148
**Development/Differentiation**		[34]	4.38 × 10^-6^ to 0.0083
GO:0048731: System development		78	4.38 × 10^-6^
GO:0048856: Anatomical structure development		81	8.42 × 10^-6^
GO:0007275: Multicellular organismal development		87	6.80 × 10^-5^
GO:0048468: Cell development		31	8.17 × 10^-5^
GO:0032502: Developmental process		91	1.82 × 10^-4^
GO:0048869: Cellular developmental process		59	5.41 × 10^-4^
GO:0030154: Cell differentiation		57	5.74 × 10^-4^
GO:0048513: Organ development		56	0.0036
GO:0045595: Regulation of cell differentiation		18	0.0083
**Gene ontology: cellular component**		**Gene count in study**	**Enrichment**^**1**^
**Extracellular region**		35	2.34 × 10^-10^ to 0.022
GO:0005576: Extracellular region		81	2.34 × 10^-10^
GO:0044421: Extracellular region part		43	2.70 × 10^-7^
GO:0031012: Extracellular matrix		22	1.16 × 10^-5^
GO:0005578: Proteinaceous extracellular matrix		21	2.14 × 10^-5^
GO:0005615: Extracellular space		24	0.0020
GO:0044420: Extracellular matrix part		7	0.0202
**Membrane association**		227	2.46 × 10^-16^ to 1.01 × 10^-6^
GO:0005886: Plasma membrane		134	2.46 × 10^-16^
GO:0031224: Intrinsic to membrane		191	1.07 × 10^-8^
GO:0044425: Membrane part		215	2.08 × 10^-8^
GO:0016020: Membrane		226	7.80 × 10^-8^
GO:0031224: Intrinsic to membrane		178	1.01 × 10^-6^

^1^P-value of enrichment of GO terms using DAVID analysis software; similar results were found with GOstat.

**Figure 4:**
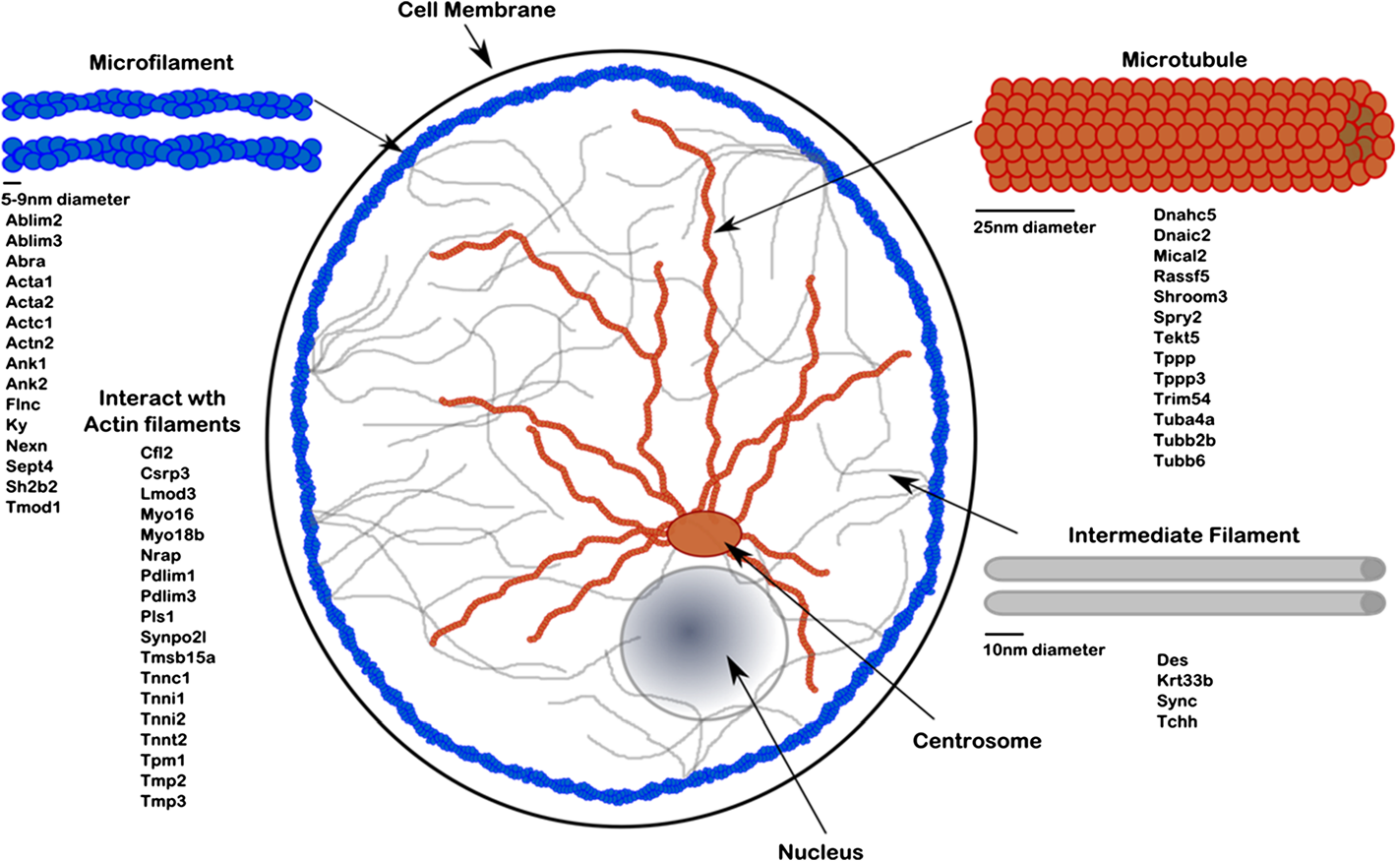
Visual representation of DE genes associated with cytoskeletal components.

110 genes associated with the Cytoskeleton were down-regulated in Pax3^*Spd/Spd*^ humeri, including those encoding microfilament, microtubule and intermediate filament components (Figure 4); 33 are directly associated with microfilaments (e.g. *Acta1, Ablim2, Pdlim3*), 13 with microtubules (e.g. *Rassf5, Tubb2b, Tppp*) and 4 with intermediate filaments (*Des, Krt33b, Sync, Tchh*); other DE genes associated with the cytoskeleton encode proteins that interact with myosin, or the extracellular matrix (ECM), including integrin and cadherin encoding genes (e.g. *Lamb3, Cdh4, Itga4 and Pdgfa* (not shown)).

### Up-regulated genes are associated with cell adhesion, cell signalling and development and differentiation

Genes up-regulated in muscle-less humeri revealed by microarray and RNA-seq were similarly analysed for enrichment of genes associated with particular biological processes or cellular components, using associated GO terms (Table 4). For terms within the subontology biological process, the strongest enrichment was for cell adhesion and ECM associated genes (total of 50/447 genes, with p-values down to 1.99 × 10^-10^). The genes identified in this category include ECM glycoproteins (*Lsamp, Svep1*), ECM structural constituents (*Col8a1, Col8a2, Frem3*), cell-adhesion molecules (*Cntn1, Cntn4, Cntn5*) and calcium-dependent cell adhesion proteins (*Pcdh8, Pcdhb2, Pcdhga1*). This grouping in addition included genes involved in signalling pathways which overlap with the next most enriched terms; cell signalling (GO:0007154) and cell-cell communication (GO:0007267). The signalling pathway components identified in this category include: Hedgehog (*Hhip, Ptch1*), fibroblast growth factor (*Fgf10, Fgf2*), transforming growth factor (*Frem2, Bmp3*), Notch (*Ctn1, Nrg1*) and Wnt signalling (*Dkk2, Cpz Rspo2, Rspo3, Sfrp2, Wnt2, Wnt2b, Wnt4* and *Wnt16*) and others, including receptors (*Epha3, Epha4, Epha5, Grin2a, Grm4, Grm7, Grm8, Gfra2,* and *Pdgfra*). Other signals identified as up-regulated included c-fos induced gowth factor (*Figf),* hepatocyte growth factor *(Hgf*) and Insulin-like growth factor (*Igf1*). The gene lists in the next most enriched set, Development and Differentiation, similarly show large overlap due again to the presence of the signalling pathway genes mentioned above, and also transcription factors *Foxc2, Foxo3, Lmx1a, Lmx1b. z.*

Under the subontology Cellular Component there was also striking enrichment of extracellular (total 81/447; with p-values down to 2.34 x10^-10^) and membrane associated gene products (total 227/447 p-values down to 2.46 × 10^-16^), including cell adhesion molecules (*Cadm2, Cntn1*), receptors (*Fat2, Fat4, Grin2a, Grm4, Gfra2, Pdgfra, Robo2, Sorcs1, Sstr4*), cell surface molecules (*Cd83, Cd96*), cadherins (*Cdh20, Cdh8*), trans-membrane proteins (*Tmem26, Tmem28*), voltage gated channels (*Kcna1, Kcna2, Kcnc2*) and cell adhesion and extracellular components: *Alcam, Cntn4, Epha4, Col8a1, Col8a2, Pappa, Pcdh8*.

### Signalling pathway analysis of differentially expressed genes

**Figure 5:**
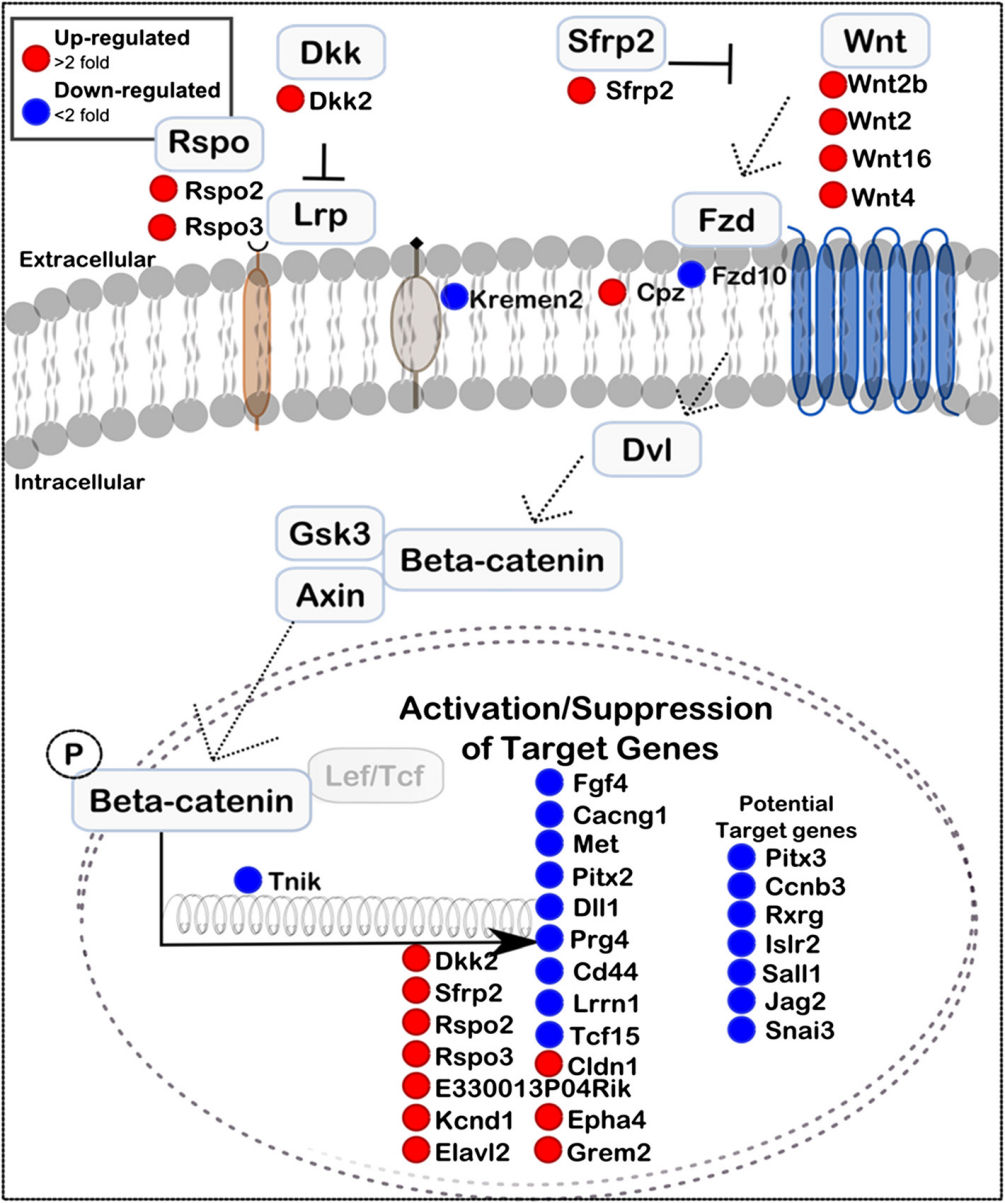
**Visual representation of Wnt signalling pathway components, showing altered expression in developing muscle-less humeri.** Blue circles indicate down-regulated genes red are up-regulated genes (Fold change ≥2, p ≤ 0.05, genes and details listed in Table 7).

Given the strong enrichment of genes associated with Signalling Pathways and Development and Differentiation functions (Tables 3 and 4), we sorted DE genes according to participation in major developmental regulatory pathways (Figure 5 and Tables 5 and 6). By far the most strongly impacted cell communication pathway is Wnt signalling with 34 DE genes encoding signalling molecules, receptors, pathway antagonists, known targets or potential targets of the pathway (Figure 5, Table 5). From the diagrammatic representation of pathway components shown in Figure 5, it is apparent that the encoded products of DE genes act either at the cell surface in Wnt signal generation/modulation/interpretation or are targets of the pathway. The genes listed include known targets of the pathway and seven potential target genes (not verified), included here due to their similarity to known targets; for example *Sall1*is included because the orthologous gene *Sall4* is a known direct target of the pathway [36]. In general, genes encoding cell surface components of the pathway are up-regulated, including signalling ligands and agonists (*Wnt2, Wnt2b, Wnt4, Wnt16, Rspo2 and Rspo3*), and extracellular antagonists (*Dkk2, Sfrp2),* while down-regulated genes identified are more commonly targets of the pathway (e.g. *Fgf4, Cacng1, Pitx2, Dll1, Prg4, Lrrn1, Met, and Cd44)*. Interestingly nine known Wnt target genes are up-regulated, including *Dkk2, Rspo2, Rspo3, Cldn1*, *Grem2, Kcnd1, Epha4* and *Sfrp2*, which encode membrane associated proteins, some of which regulate the Wnt pathway (*Dkk2, Sfrp2, Rspo2, Rspo3).*

**Table 5:** Differentially expressed Wnt signalling pathway genes

**Down-regulated Wnt Pathway genes**								
**Fold change**		**Gene symbol**	**Gene title**	**Role in pathway**				**Reference for role in pathway**
**RNA-seq**	**Microarray**			**Ligand**	**Receptor**	**Intra-cellular**	**Target**	
#	41.05	Fgf4	Fibroblast growth factor 4				T	[37]
#		Snai3	Snail homolog 3				PT	Snai1 [38]
90.87	32.06	Cacng1	Calcium channel, voltage-dependent, gamma subunit 1				T	[39]
42.86	18.77	Pitx3	Paired-like homeodomain transcription factor 3				PT	Pitx2 [40]
37.43	17.22	Kremen2	Kringle containing transmembrane protein 2		Co-Rec			[41]
29.52		Ccnb3	Cyclin B3				PT	CyclinD1 [42]
12.29	7.05	Rxrg	Retinoid X receptor gamma				PT	Rarg [43]
5.95	5.20	Islr2	Immunoglobulin superfamily containing leucine-rich repeat 2				PT	Islr [44]
4.87		Pitx2	Paired-like homeodomain transcription factor 2				T	[40]
3.58		Fzd10	Frizzled homolog 10		R			[45]
3.16	2.21	Dll1	Delta-like 1				T	[46]
3.05		Prg4	Lubricin				T	[30]
2.81	2.57	Lrrn1	Leucine rich repeat protein 1, neuronal				T	[47]
2.52		Sall1	Sal-like 1				PT	Sall4: [36]
2.47		Tnik	TRAF2 and NCK interacting kinase			TF-act		[48]
2.32		Tcf15	Transcription factor 15				T	
2.32		Met	Met proto-oncogene				T	[49]
2.18	2.28	Cd44	CD44 antigen				T	[50]
2.09		Jag2	Jagged2				PT	Jag1 [51]
Up-regulated Wnt pathway genes								
4.36	4.91	Grem2	Gremlin				T	[52]
3.96		Wnt2	Wingless-related MMTV integration site 2	L				
3.50		Wnt2b	Wingless-related MMTV integration site 2b	L				
3.07		Wnt16	Wingless-related MMTV integration site 16	L				
2.85	2.90	Kcnd2	Potassium voltage-gated channel, shal-related family, member 2				T	[47]
2.73		Cldn1	Claudin-1				T	[53]
2.44		Dkk2	Dickkopf	Ant			T	[47,54]
2.43	3.08	Rspo2	R-spondin 2	L			T	[47,55,56]
2.37		Epha4	Eph receptor A4				T	[47]
2.11		Elavl2	ELAV (embryonic lethal, abnormal vision, Drosophila)-like 2				T	[47]
2.10		Cpz	Carboxypeptidase Z	Co-act				[33]
2.09	2.62	Sfrp2	Secreted frizzled related protein 2	Ant			T	[57]
2.03		Wnt4	Wingless-related MMTV integration site 4	L				
2.02		Rspo3	R-spondin 3	L			T	[47,58]
	2.41	E330013P04Rik	RIKEN cDNA E330013P04 gene				T	[47]

#No numerical value for fold change as no transcripts detected in the Mutant.

T -Target, PT -Potential Target, R -Receptor, Co-Rec -Co-Receptor, TF-act -Transcription Factor activator, L -Ligand, Ant -Antagonist, Co-act -Co-activator.

**Table 6:** Differentially expressed genes associated with other signalling pathways

**Down-regulated**			**Up-regulated**		
**Bone morphogenetic protein signalling pathway**					
**Fold change**		**Gene symbol**	**Gene title**	**Role in pathway**	
**RNA-seq**	**Microarray**				**Reference**
87.94	50.88	Hfe2	Hemochromatosis type 2	Co-Rec	[59]
5.25		Fgf8	Fibroblast growth factor 8	T	[60]
2.44		Scx	Scleraxis	T	[61]
2.17		Egr2	Early growth response 2	T	[62]
2.03		Bmp3	Bone morphogenetic protein 3	L	
Hedgehog signalling pathway					
14.55	4.59	Hhatl	Hedgehog acyltransferase-like	L-act	[63]
2.70		Ptchd1	Patched domain containing 1	R	
2.27		Hhip	Hedgehog interacting protein	Ant, T	[64]
2.14		Ptch1	Patched 1	T	[65]
Fibroblast growth factor signalling pathway					
#		Fgf4	Fibroblast growth factor 4	L	
#		Fgf6	Fibroblast growth factor 5	L	
5.25		Fgf8	Fibroblast growth factor 8	L	
5.10		Fgf5	Fibroblast growth factor 5	L	
4.54	3.23	Fgfr4	Fibroblast growth factor receptor 4	R	
3.24		Spry1	Sprouty homolog 1	Ant	[66]
3.22		Spry4	Sprouty homolog 4	Ant	
2.75		Spry2	Sprouty homolog 2	Ant	[66]
2.39		Fgf2	Fibroblast growth factor 2	L	
2.134	2.48	Fgf10	Fibroblast growth factor 10	L	
Hippo signalling pathway					
98.29	49.05	Vgll2	Vestigial like 2 homolog (Drosophila)	Co-TF	[67]
3.86	4.38	Tead4	TEA domain family member 4	Co-TF	[68]
2.96		Fat4	Fat tumor suppressor homolog 4	R	[69]
2.43		Fat2	Fat tumor suppressor homolog 2	R	[69]
Notch signalling pathway					
5.64		Dner	Delta/notch-like EGF-related receptor	R	[70]
3.16	2.21	Dll1	Delta-like 1 (Drosophila)	L	[71]
2.69		Hes6	Hairy and enhancer of split 6	T	[72]
2.54		Dtx4	Deltex homolog 4	T	[72]
2.09		Jag2	Jagged 2	R	[71]
3.25	2.87	Nrg1	Neuregulin 1	T	[73]
2.36	2.09	Foxc2	Forkhead box C2	Upstream-activator	[74]
2.27	2.29	Cntn1	Contactin 1	PL	[75]

#No numerical value for fold change as no transcripts detected in the Mutant.

Co-Rec -Co-Receptor, T -Target, L -Ligand, L-act- Ligand activator, R -Receptor, Ant -Antagonist, Co-TF- -Co-Transcription Factor, PL –Potential Ligand.

Table 6 lists DE genes associated with other developmentally relevant signalling pathways, including the BMP, Hedgehog, Fibroblast growth factor, Hippo and Notch signalling pathways.

### Spatial alteration of gene expression patterns

**Figure 6:**
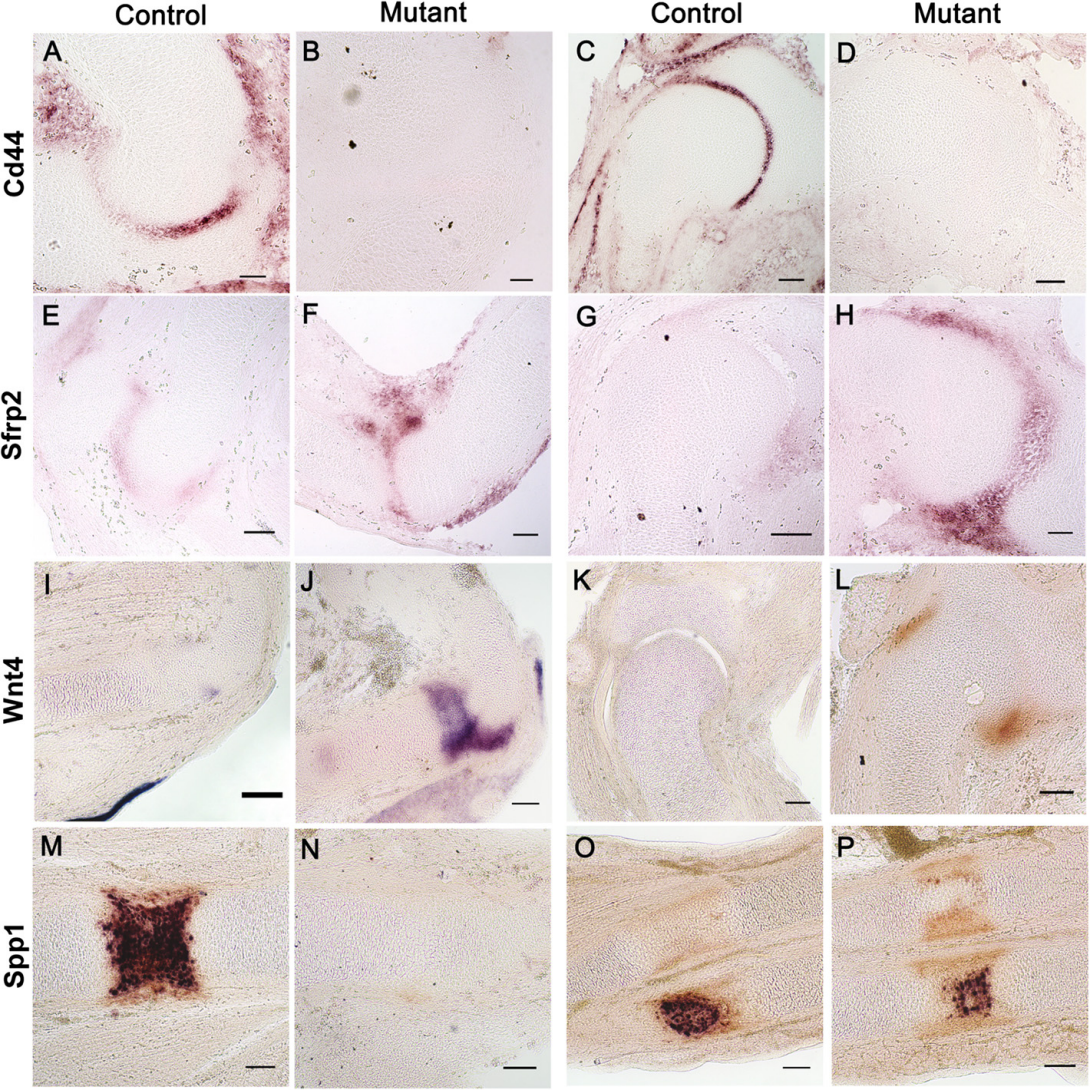
**Comparison of spatial distribution of differentially expressed genes in TS23 control (A,C,E,G,I,K,M) and TS23 mutant (B,D,F,H,J,L,N,P) elbow joint, shoulder joint, humerus, radius and ulna.***Cd44***(A-D)** expression in the elbow joint **(A-B)**, and the shoulder joint **(C-D)** (arrows in **B** and **D** indicate the position of joint-line cells based on cell morphology), *Sfrp2***(E-H)** expression in the elbow joint **(E-F)** (arrow indicates abnormal expression on the ventral side of the joint), and the shoulder joint **(G-H)**, *Wnt4* expression in the elbow joint **(I-J)**, and the shoulder joint **(K-L)**, *Spp1***(K-N)** expression in the humerus hypertrophic zone (hz) and perichondrium (p) **(M-N)** (the arrowhead in **L** indicates enlarged hypertropic chondrocytes), and in the ulna (u) and radius (r) **(O-P)**. e epidermis. All scale bars are 100 μm.

While Microarray and RNA-seq analysis provides data on quantitative changes in gene expression levels across the whole developing rudiment, it does not reveal alterations in the spatial distribution of transcripts or give clues to the specific developmental events affected. We therefore performed in situ hybridisation on control and muscle-less mutant (*Pax3*^*Spd/Spd*^*)* limb sections at TS23 for a selected subset of genes. Three of the genes selected encode components of Wnt signalling pathways known to be important in skeletal development: *Cd44, Sfrp2* and *Wnt4. Spp1* encodes an ECM protein Osteopontin which is a prominent component of mineralised matrices of bone and teeth [76]. Cd44 is a cell-surface glycoprotein involved in cell-cell interactions, cell adhesion and migration. It is a receptor for hyaluronic acid and can interact with other extracellular proteins, such as osteopontin, collagens and matrix metalloprteinases (MMPs) reviewed in [77]. It is a target gene of the Wnt signalling pathway [50]. *Cd44* gene expression is down-regulated 2.28 fold in muscle-less humeri RNA (Table 5). The in situ hybridisation analysis reflects this down regulation dramatically with Cd44 transcripts hardly detectable in either shoulder or elbow joints of muscle-less mutant embryos, compared to the clear joint line restricted expression seen in controls (Figure 6A-D). Sfrp2 encodes a secreted protein that acts as a modulator of the Wnt signalling pathway, in particular during normal skeletal patterning in developing limbs [78]. Normal expression at TS23 (Figure 6E, G) can be detected in the elbow and shoulder joints. The level of up-regulation from microarray and RNA-seq analysis is 2.62 (p-value 0.01) and 2.09 (p-value 1.13E-06) fold respectively (Table 5). This up-regulation was reflected in the intensity of expression seen in mutant sections following in situ hybridisation (Figure 6F, H). In addition to the increased level of expression a change in the spatial pattern is also evident. In both the elbow and the shoulder joints expression is expanded, particularly on the ventral aspect of the joint and the staining is unevenly distributed (Figure 6F, H). A similar expression increase was seen in the phalangeal and carpal joints of the handplate, compared to control (not shown). Wnt4 is a signalling ligand of the Wnt signalling pathway. Expression of this gene has previously indicated its role in joint formation during limb development [79-81]. Control *Wnt4* expression is seen at a low level in the ventral portion of the elbow joint (Figure 6I) at TS23; there is also distinct expression in the epidermis, as previously detected [82]. In the mutant there is a high level of expression in the elbow joint region; expression is spread across the whole joint line from ventral to dorsal, more extensive on the dorsal side (Figure 6J).

The *Spp1* gene is normally expressed in the hypertrophic zone and adjacent perichondrium [83]. Despite the appearance of hypertrophic chondrocytes at the mid-diaphysis of immobile *Spd* embryos (Figure 6N arrowhead), no *Spp1* gene expression is detected in these cells. There is weak but detectable staining in the perichondrium at the site of the hypertrophic region, but again apparently lower than in control tissue (Figure 6M, N). Expression is also detected in the hypertrophic zone of the ulna (Figure 6O) and this is reduced but not absent in the muscle-less mutant ulna (Figure 6P), reflecting the reduced phenotypic effect seen in this rudiment [9]. Expression is seen only in the perichondrium of the radius in both the control and mutant (Figure 6O, P).

## Discussion

Here we describe the spectrum of genes expressed in the developing humerus at TS23, early in the process of ossification and when territories of differentiating cells are being defined in the developing joint region. We also use microarray and RNA-sequencing to identify genes that are differentially regulated when mechanical stimulation of the developing skeletal rudiment is altered, giving an insight into the genes that respond to mechanical stimuli generated by muscle contractions. We reveal that the genes altered are highly enriched for genes that regulate development and differentiation, are involved in cytoskeletal rearrangement and components of extracellular matrix including cell adhesion and signalling molecules. Components of multiple signalling pathways important during development are affected, in particular 34 components of the Wnt signalling pathway. Although it is clear that appropriate mechanical stimulation from *in utero* muscle contractions is required for normal development of bone and cartilage, we know very little about the molecular mechanisms that incorporate mechanical cues with classical biochemical signalling pathways. The differentially regulated genes identified here, particularly those associated with signalling pathways and cytoskeletal changes represent a valuable focus for dissecting integrated regulation by biochemical and mechanical signals. These data represent an important resource that can be utilised to understand the molecular basis of mechanoregulation.

### The transcriptome of a developing skeletal rudiment

Utilising RNA-sequencing technology to reveal the transcriptome in the normal developing humerus and associated joints at TS23 provides an insight to the processes that are occurring during this stage of skeletal development when chondrocytes are undergoing hypertrophy, the cartilaginous rudiment is beginning to ossify at the mid diaphysis reviewed in [84] and specific zones within the joint are differentiating reviewed in [85]. This adds a valuable resource to a growing set of data that can be combined to explore skeletal development. Previous transcriptome profiling studies have examined cartilage condensation in the tibia-fibula from E11.5 to E13.5 [86], ossification of metatarsals [87], in different zones of the growth plate in 2 week old mice [88] and in Runx2^-/-^ mutant mice [89]. Earlier stages of limb development, although not specific to skeletal development, are informed by whole limb bud profiles between E9.5 and E11.5 [32]. Another important resource that has recently become available examines the spatial expression patterns of 18,000 coding genes and over 400 microRNAs within the whole mouse embryo at the same developmental stage examined here [90]. This digital transcriptome atlas is a powerful tool (Eurexpress; http://www.eurexpress.org/ee/intro.html) that can be used to examine spatial analysis of specific genes, exploring possible functional associations. Combining these resources gives information on quantitative and spatial expression of individual genes providing the basis to explore regulatory networks active during the development of skeletal rudiments.

Several of the findings of the transcriptome analysis are as expected; the most highly expressed genes (8 with ≥100,000 read counts) include 5 collagen encoding genes (Figure 2B); collagens have been shown to be the most abundant structural proteins in cartilage and show characteristic distribution patterns as skeletal rudiments develop [31,91]. The Insulin-like growth factor genes *Igf2* and *Igf1* and their associated receptors *Ifg2r* and *Igf1r* are also highly expressed (4,194 to 221,194 read counts); these are reported to play a prominent regulatory role in skeletal development (reviewed in [92]). Similarly, aggrecan (*Acan)* and osteopontin (*Spp1*), both involved in skeletal development, are highly expressed (Figure 2B). Although much is known about the regulatory network that controls early chondrogenesis and joint formation (Kronenberg [25]; Provot and Schipani [26]), open-ended whole transcriptome studies are required to add new information. Centred on regulatory signalling pathways known to be involved in skeletal development; HH, FGF, TGFβ (including BMP) and Wnt; a complete list of components of these pathways expressed in the humerus as TS23 was extracted (Figure 2B) indicating the potential role players in each of these pathways at this specific stage. The importance of Indian hedgehog (*Ihh)* expression in the early prehypertrophic and hypertrophic chondrocytes of cartilage condensations is well established [89,93,94]. The full spectrum of possible interacting molecules in HH signalling (Figure 2B) include the receptors *Ptch1, Ptch2, Smo* and transcription factors *Gli1, Gli2,* and *Gli3.* In addition to *Ihh,* Desert hedgehog (*Dhh*) expression was also detected (read count 70) and, examining the data presented by Cameron et al. confirms that Dhh is up-regulated (≥3 fold) in E13.5 fibual and tibual cartilage [86]. *Dhh* has not previously been functionally linked to skeletogenesis but this opens the possibility of regulatory contribution, perhaps co-operating with Ihh. No expression of *Shh* was detected. Similarly, novel components of the FGF, TGBβ and Wnt pathways were identified (Figure 2B).

The Wnt signalling pathway plays a central role during embryonic development and is known to be an important regulator of bone formation and bone remodelling reviewed in [95]. It also plays a pivotal role in joint formation and maintenance, shown through gain and loss of function experiments [80,96]. The key intracellular mediator of canonical Wnt signalling, β-catenin (*Ctnnb1*; 28,608 read counts) is the most highly expressed Wnt signalling component in the TS23 humerus and associated joints. The most highly expressed Wnt ligand is *Wnt5a*, previously associated with expression in joints and perichondrium [79] and proliferating chondrocytes [97]. Other highly expressed ligands include *Wnt9a, Wnt5b*, *Wnt11,* and *Wnt4. Wnt5b* and *Wnt11* expression has been shown in the pre-hypertrophic chondrocytes and *Wnt4, Wnt9* and *Wnt16* in the developing joints [79-81,98]. High expression of *Wnt9a* could be due to its role in the temporal and spatial regulation of *Ihh*[96].

Numerous extracellular modulators of the pathway were detected; all five secreted frizzled related protein (*Sfrp)* genes, Dickoff (*Dkk)* 1, 2 and 3 genes and four R-spondins (*Rspos)*, indicating a huge potential for pathway modulation. The most highly expressed antagonist modulators of the pathway were *Dkk3* and *Sfrp2* both of with are detected in joint cells at E13.5 and E15.5 [82]. The most highly expressed R-spondin agonist of the pathway was *Rspo3*, previously detected in phalanges [55].

### Identification of Mechanoresponsive genes

The identification of differentially expressed genes between humeri from control and muscle-less embryonic limbs allows an investigation of the biological processes and the developmental regulatory signalling pathways that are affected by the removal of mechanical stimulation on skeletogenesis *in vivo*. We previously reported that muscleless (*Splotch*) mutants display abnormal ossification in the humerus, altered humeral morphogenesis and altered elbow and shoulder joint formation and these effects were first observed at TS23 [9]. This was chosen as the point of analysis for differential gene expression because, although limb muscles begin to contract from approximately E12.5 [99] it is uncertain how much stimulation is transmitted to the skeletal rudiments when the developing tendons are at early stages of morphogenesis [100]; the sole indication that the force is functionally transmitted is the mutant phenotype seen at TS23. Although analysis at TS23 may miss some of the earliest effects, it is relatively early in the response and the earliest time at which it is certain that the system is disturbed. Alteration in expression pattern of some selected candidate genes and pathways was previously revealed [8,10], but here we carry out the first genome wide study identifying a total of 1,132 independent genes as differentially expressed: with approximately 60% down-regulated and 40% up-regulated. The finding of more genes being down-regulated than up-regulated and to a greater extent is consistent with the proposal that mechanical stimuli support the correct differentiation of cells, as observed in the ossification phenotype [9], and for the maintenance of tissue patterning, as seen in the developing joint [16]. GO annotation analysis identified specific biological processes that are affected when mechanical stimuli are removed. This type of analysis has been used previously to interpret biological processes associated with developing skeletal tissue [87,89,101]. Analysis of the down-regulated DE gene set identified genes associated with development and differentiation as the most highly enriched categories, including developmental regulatory signalling pathway molecules and transcription factors. Similarly, analysis of up-regulated DE gene sets indicated genes associated with cell signalling and development and differentiation.

DE genes were also highly enriched for genes associated with the cytoskeleton. The cytoskeleton controls cell shape, organelle transport, cell motility and division, and connects the extracellular matrix to internal cell processes reviewed in [102]. It maintains the mechanical integrity of cells and has been implicated in relaying mechanical signals to downstream biochemical responses [7,103]. This was seen in the embryonic lung where cytoskeletal network inhibitors resulted in altered tissue morphogenesis and conversely when cytoskeletal tension was activated lung development was accelerated reviewed in [104], indicating the dynamic role the cytoskeleton has in morphogenesis.

In chondrocytes the actin microfilaments are predominantly located at the periphery of the cytoplasm [105], tubulin microtubules are uniformly distributed throughout the cytoplasm [106] as are intermediate filaments, connecting the nuclear membrane with the cell periphery [107]. In this study 84 genes annotated as cytoskeletal were down-regulated when mechanical stimulation was removed. These include 33 genes directly associated with actin microfilaments, 13 with microtubules and 4 with intermediate filaments (Figure 4). The most highly affected group, the Filamentous-actin cytoskeleton, has been shown to be involved in articular cartilage chondrocyte mechanotransduction, converting a mechanical stimulus into a biochemical response [103,108-110]. Other studies have confirmed the involvement of the actin cytoskeleton in cartilage chondrocyte mechanotransduction via manipulation of the actin accessory proteins [108,111], but there are few reports on the affect of mechanical stimulation on microtubule and intermediate filaments [112]. Among the DE genes is an actin-binding protein, cofilin2 (*Cfl2*); cofilin was previously shown to be increased following cyclic mechanical loading of chondrocytes [111].

The identification of cytoskeletal genes down-regulated following the removal of mechanical stimulation indicates that the cytoskeleton is affected, but is this because the mechanical integrity of the cell is altered or because mechanotransduction from the ECM is affected, or perhaps a combination of both? The finding that ECM and cell adhesion associated genes are also affected further supports changes in mechanotransduction pathways. The cell-adhesion associated integrins (*Itga4*, *Itga7, Itgb6*) and cadherins (*Cdh4, Cdh15, Cdhr3*) are down-regulated and these proteins potentially function to physically couple cells to the ECM and play a role in mechanical signal transduction [103,104]. Articular chondrocytes have been shown to express both integrin [113] and non-integrin [114] ECM receptors. Another actin-associated protein identified to be down-regulated is actinin-α2 (*Actn2*); this protein also couples the cytoskeleton to the ECM and may be involved in transducing mechanical stimulation.

Secreted phosphoprotein 1 (*Spp1*), previously known as Osteopontin (Opn), is one of the abundant non-collagenous proteins in bone matrix produced by osteoblasts and osteoclasts reviewed in [115]. Spp1 binds to hydroxyapatite and is a potent inhibitor of the mineralisation process, inhibiting the growth of bone matrix crystals [116]. Spp1 is expressed early in bone development, however it was concluded not to be required for normal development of bones as null mice (OPN^-/-^) have no apparent effect on the structure or distribution of cells within bone tissue [117]. However, Spp1 expression has been shown to be regulated by mechanical stimulation both *in vitro* and *in vivo*[35,118-120]. We found *Spp1* to be down-regulated in the developing humerus at TS23 in muscle-less embryos and in situ hybridisation showed a dramatic absence of detectable Spp1 expression in hypertrophic chondrocytes whereas it is still detectable in the perichondrium (Figure 6N), indicating a specific effect on expression in hypertrophic chondrocytes and not a delay in the onset of normal expression. It was previously shown that OPN^-/-^ mice did not suffer bone loss in response to mechanical unloading [120], suggesting that mice lacking Spp1 could not sense the changes in mechanical stress, thus indicating its potential role in the signal transduction of mechanical stimulation. It has been suggested that mechanotransduction through Spp1 is dependent on microfilament integrity, as mechanically stimulated increases in *Spp1* expression was blocked by disruption with cytochalasin-D in osteoblasts [119]. This again highlights the link between an ECM component and the cytoskeleton in a mechanoresponse implicating these components in signal transduction, either directly through the cytoskeleton or through cell adhesion complexes via the cytoskeleton.

An example of a non-integrin ECM component that is down-regulated in the absence of mechanical stimulation is Cd44, a target gene of the Wnt signalling pathway [50], encoding a single-pass membrane glycoprotein that binds proteoglycan and hyaluronan (HA) to produce a pericellular matrix surrounding chondrocytes reviewed in [121]. Cd44 has been implicated in joint cavitation through interaction with HA [122]. We previously showed loss of expression of the Cd44 gene in the interzone of the forming knee joints of immobilised chick embryos [16], one of a number of gene expression patterns reflecting a general loss of organisation of differentiating tissue territories, and here we show a similar effect on Cd44 expression in the elbow and shoulder joint of muscle-less mouse limbs, where we previously showed a similar loss of tissue organisation [9]. The very restricted expression of Cd44 in the interzone of forming joints in control embryos at TS23 is barely detectable in muscle-less mutants (Figure 6A-D). As well as the gene being sensitive to mechanical stimulation, as an integral part of the ECM and a regulator of joint formation, the gene product may also be an important mediator of mechanical stimuli.

The link between Cd44 and the Wnt signalling pathway highlights perhaps the most striking finding of this analysis; the altered expression of 34 genes implicated in the Wnt signalling pathway (Figure 5). Canonical Wnt signalling has been shown to be involved in maintaining joint integrity [80,96] and is disturbed in the joints of muscle-less mouse embryos [8]. Wnt signalling might also be involved in co-ordinating ossification and joint development; both processes altered in muscle-less embryos [5]. Non canonical signalling has also been implicated in planar cell polarity during growth plate regulation [123,124]. There are also previous indications that the Wnt pathway is responsive to mechanical stimulation in mesenchymal stem cells [125] in mature bone *in vivo*[126] and in response to injury of articular cartilage [127]. Here, genes encoding four Wnt ligands are up-regulated in muscle-less embryos and in the case of *Wnt4* we show specific up-regulation in the elbow and shoulder joint region (Figure 6I-L). Two of the up-regulated genes (*Wnt2* and *Wnt2b*) have not previously been associated with skeletal development. The Wnt signalling antagonist *Sfrp2* is also up-regulated specifically in the joint region (Figure 6E-H). The majority of known Wnt target genes affected are down-regulated (Figure 5), perhaps due to increased expression of negative regulators *Sfrp2* and *Dkk2* and down-regulation of the *Fzd10* receptor; however ligands and agonists *Rspo2* and *Rspo3* are up-regulated as are some target genes indicating effects at multiple levels of regulation of the pathway. It is interesting that a number of the up-regulated targets feedback as negative regulators of the pathway (for example: *Dkk2*, *Sfrp2*). It is now important to functionally test the mechanisms linking mechanical stimulation with Wnt signalling. This work provides sets of candidate genes to use in functional assays to excavate this important link. Understanding how mechanical stimuli influence the Wnt signalling pathway would be a major step forward in understanding how mechanical cues work together with classical molecular positional information to guide spatially appropriate tissue differentiation and provide indications of how conditions can be effectively recreated in vitro to guide stem cell differentiation.

In situ hybridisation analysis showed altered gene expression of 3 Wnt pathway genes in the developing shoulder and elbow joints; one down regulated (Cd44) and two upregulated (Sfrp2 and Wnt4) (Figure 6). Changes in the spatial restriction of Sfrp2 and Wnt4 expression were also seen. This does not represent a delay in normal expression in the mutant because the altered pattern is not reminiscent of earlier stages [82] and the changes are consistent with altered patterning of the tissue territories in the forming joint and the fusion phenotype seen in the mutant, with cartilage forming across the joint at later stages [8,9].

In this study differential expression in developing skeletal rudiments is documented in the absence of limb muscle; this will include genes that respond to lack of mechanical stimulation but perhaps also as a paracrine response to adjacent muscle cells. We know that phenotypic effects on ossification and joint formation are due to the lack of mechanical stimulation rather than physical absence of muscle cells because phenotypic analysis of a range of mouse mutants where muscle is immobile [8], reduced [9] or absent [8,128] have similar effects and we see similar effects in immobilised chick embryos [15,16]. Therefore, although some of the genes identified here may respond to lack of adjacent muscle tissue, many must underlie the phenotypic effects seen in response to lack of mechanical stimulation. This is further supported by the overlap of some of the genes identified here and in skeletal cells in culture or adult tissues, in response to mechanical stimulation [18,19].

Another important limitation in this work is the possibility that a proportion of the down-regulated genes may be due to contamination of the dissected control humeri with adjacent mesenchyme/muscle, since this is being compared to tissue from muscle-less embryos. Although care was taken with the dissections, it is impossible to be sure that all muscle tissue was eliminated from the control. The down-regulated gene set also showed enrichment for muscle associated genes (Additional file 1: Table S4), consistent with possible contamination of the dissected control humeri by neighbouring muscle. To inform this we also sequenced the transcriptome of mesenchyme adjacent to the humerus of control embryos at TS23 and compared it to the transcriptome of control humeri. We then cross referenced this to the down-regulated gene set in control versus muscle-less humeri (Additional file 1: Table S2), noting any genes enriched more than 3 fold in mesenchyme compared to control humeri; these are indicated in column 2 of Additional file 1: Table S2. It is possible that these genes are involved in both cartilage and muscle development so no genes have been removed from the data set, however, DE genes also showing higher expression in mesenchyme compared to control humeri must be treated with caution with respect to a skeletal specific response to mechanical stimulation. Such genes have not been prioritised in any of our subsequent exploration of candidate mechanosensitive genes.

The developing humerus at TS23 constitutes different cell and tissue populations at different stages of differentiation including the joint region, the perichondrium and the organised zones within the cartilage rudiment. Therefore the experimental design employed here will capture genes associated with different cells types at different stages of differentiation. It will now be important to sort out which cells and tissues have altered expression of specific genes. This can be addressed for a sub set of genes by in situ hybridisation, with an initial analysis of 4 genes presented in Figure 6. It can be addressed in a high throughput manner by isolating specific cell populations using laser microdissection from tissue sections (laser capture), purification of RNA and quantitative RT-PCR gene expression profiling, comparing control and mutant tissue from,, for example the hypertrophic, prehypertrophic or the elbow joint region alone.

We used both RNA-sequencing (Illumina) and Microarray (Agilent) technologies in parallel to determine differential expression. Microarray technology has been utilised to determine expression of chondrogenic and osteogenic genes from developing whole tissues [32,87,89], and from *in vitro* differentiation procedures [19,34,129-131]. The use of RNA-seq technology to describe the transcriptome is more recent [132-134]. Previous direct comparisons between microarray and RNA sequencing-based approaches to reveal alterations in gene expression between tissues reported that RNA-seq identified more DE genes [23,24,135]. We also found that RNA-seq is more sensitive in reproducibly detecting alterations in gene expression, detecting more genes altered at lower quantitative levels (Additional file 1: Table S1; 5 > 2 fold). This was further emphasised by reducing the stringency of the statistical analysis to p ≤ 0.08, which increased the number of genes detected by microarray specifically (not shown). An example of the importance of the increased sensitivity and reproducibility of RNA-seq is shown by the *Spp1* gene which did not show statistical significance by microarray but has been verified by qRT-PCR and in situ hybridisation (Table 2 and Figure 6). The larger dynamic range [24] and higher reproducibility across replicates [135] has also been found in other studies.

## Conclusion

This study examines the set of genes active at a key stage of skeletal development (TS23) and reveals the genes that are differentially regulated in the developing humerus when skeletal muscle is absent. Since we previously showed that the lack of muscle contractions leads to common phenotypic defects in both ossification and joint formation in several chick and mouse models, this provides an insight into the genome wide alterations in gene transcription that take place when the mechanical environment is altered. Given the importance of appropriate mechanical stimulation generated by embryo movement on skeletal development we postulated that mechanical stimuli must integrate with biochemical cell signalling pathways known to be essential for normal development. We show that multiple signalling pathways are affected, with components of the Wnt signalling pathway most strongly disturbed including 4 Wnt ligands and both down-regulation and up-regulation of target genes. Down-regulated genes include *Cd44*, *Dll1* and *Fgf4* which are involved in further cellular interactions during joint formation or feed into other important cell communication events. Among the up-regulated Wnt targets are several genes that feed back into the Wnt pathway itself as antagonists (*Sfrp2* and *Dkk2*) or agonists (*Rspo2, Rspo3*). This finding, together with alteration of cytoskeletal components, indicates the biological processes involved in integrating biophysical stimuli during cell differentiation and patterning. Understanding the mechanistic basis for how developing cells interpret and respond to biophysical cues is a major challenge, relevant to all developing systems, and will impact our ability to control differentiation of progenitor cells for regenerative therapies. This work is an early step in unravelling the mechanistic basis of biophysical regulation of skeletal development and provides a focus for future studies.

## Methods

### RNA preparation

Heterozygous *Splotch-delayed* (*Pax3*^*Spd/+*^) [12] mice were purchased from Jackson Laboratories (Jax®). All animal work was carried out under the guidelines of Trinity College Dublin Bioresources Unit and Bioethics Committee. The generation of homozygous *Pax3*^*Spd/Spd*^ mutant embryos was achieved by crossing heterozygous *Pax3*^*Spd/+*^ males and females. Embryonic material was collected from timed pregnancies on the afternoon of the 14th day (E14.5). Individual embryos were dissected and the developmental stage according to Theiler criteria [21], and the phenotype were recorded. All embryos were genotyped following PCR amplification as described in [136]. The humeri, including the associated joint regions, were finely dissected from control and mutant embryos at stage TS23 (Figure 1A). Tissue was mechanically homogenised and total RNA extracted (SV Total RNA Isolation System: Promega, UK). Pooling of rudiment tissue from multiple embryos of the same genotype (2–4) was performed. RNA integrity was assessed on a 2100 Bioanalyser (Agilent Technologies); RNA samples with RIN (RNA integrity number) values of 8.2-9.6 were used for Microarray and RNA-seq analysis.

### Microarray

Four independent pooled sets of samples were used for microarray (n = 4 biological replicates) analysis. All microarrays were processed at IMGM® Laboratories (Martinsried, Germany). 100 ng of total RNA per sample was reverse transcribed into cDNA and then converted into labelled cRNA by *in vitro* transcription incorporating cyanine-3-CTP (Low input quick-amp labelling kit one-colour, Agilent Technologies). Genome wide expression profiling was carried out using the Agilent Mouse GE v2 Microarrays (4x44K format) (G4846A, Agilent Technologies) which contains 39,485 coding and non-coding sequences of the mouse genome (Figure 1B). A one-colour based hybridisation protocol was performed at 65°C for 17 hours on separate mouse GE v2 microarray platforms. Microarrays were then washed with increased stringency using Gene Expression Wash Buffers (Agilent Technologies) followed by drying with Acetonitrile (Sigma). Fluorescent signal intensities were detected with Scan Control A.8.4.1 software (Agilent Technologies) in the Agilent DNA microarray scanner and extracted from the images using Feature Extraction 10.7.3.1 software (Agilent Technologies). The software tools Feature Extraction 10.7.3.1, GeneSpring GX 11.5.1 and Spotfire Decision Site 9.1.2 (TIBCO) were used for quality control and statistical data analysis. Quantile normalisation was applied to each data set in order to impose the same distribution of probe signal intensities for each array [137], thus adjusting them to a uniform level that can allow for comparable downstream analysis. Welch’s approximate t-test (“unpaired unequal variance”, parametric) was applied to compare the control and mutant groups. A corrected p-value was calculated based on the algorithm of Benjamini and Hochberg [27], based on control of the False Discovery Rate (FDR). A fold change of ≥ 2 and FDR-adjusted p-value of ≤0.05 were used as criteria to indicate differential expression between the two groups.

### RNA-sequencing: alignment and differential expression analysis

Three independent pooled sets of samples were used for RNA-seq (n = 3 biological replicates) analysis. The DNase-treated RNA (3 μg) was used to prepare RNA-Seq libraries with the TruSeq RNA Sample Prep kit. A total of six cDNA libraries were constructed, representing triplicate biological replicates for each group. 40 bp single end reads were obtained from an Illumina GAII in FASTQ format, one sample per sequencing lane. The Tophat aligner (http://tophat.cbcb.umd.edu/) was used to align the reads to the mouse reference genome (mm9). After alignment the read counts for each gene were extracted using htseq-count (http://www-huber.embl.de/users/anders/HTSeq/) based on an mm9 Refseq gff file. Differential expression in our two groups was evaluated using DESeq version 1.4.1, implemented in R 2.14.1. DESeq uses a negative binomial distribution to model genic read counts following normalisation based on size factors and variance. As for the microarray analysis, p-values were adjusted by the procedure of Benjamini and Hochberg to control the type I error rate, and a cut off of p ≤ 0.05, and a fold change of ≥ 2 were used as a threshold to define differential expression.

### Quantitative real-time reverse transcription-polymerase chain reaction

Quantitative real-time reverse transcription-polymerase chain reaction (qRT-PCR) was used to verify the relative gene expression changes in nine genes indicated to be differentially expressed (DE) by microarray and RNA seq analysis; *Fgf4*, *Cilp*, *Rxrg*, *Dll1, Spp1 Vstm2a*, *Figf*, *Fgf10* and *Sfrp2* (Table 7). All primers were designed using Primer Express Software®, version 3.0, under default settings for TaqMan® quantification and purchased through Sigma (Sigma-Aldrich, UK). Primers sets were designed with a primer Tm range of 58°-60°, an optimal length of 20 bp and an amplicon range of 50–150 bp. Total RNA was reverse transcribed (100 ng) into cDNA using iScript™ cDNA systhesis kit (BioRad) as per manufacturer’s instructions. SYBR green gene expression quantification was performed using QuantiTect SYBR green kit. 5 μl of cDNA preparation was diluted 1:5 with RNase free water, 10 μl of 2x QuantiTect SYBR green PCR master mix, 0.5 ul (10 μM) of each primer and 4ul RNase free water). Samples were assayed in triplicate in one run (40 cycles), which was composed of three stages, 95°C for 10 min, 95°C for 15 s for each cycle (denaturation) and 60°C for 1 min (annealing and extension). Real-time PCR was performed using an ABI 7500 Sequence Detection system (Applied Biosystems). qRT-PCR data was analysed using relative quantification and the C^t^ method as described previously [138], with the *Gapdh* gene as the endogenous control. The level of gene expression was calculated by subtracting the averaged C^t^ values (C^t^ is the threshold cycle) for *Gapdh* from those of the gene of interest. The relative expression was calculated as the difference (ΔΔC^t^) between the C^t^ of the test sample (mutant) and that of the control sample. The relative expression of genes of interest were calculated and expressed as 2^-ΔΔCt^. Relative quantification values are presented as fold changes plus/minus the standard error of the mean relative to the control group, which was normalised to one.

**Table 7:** Primer sequences for qRT-PCR analysis of differentially expressed genes

**Gene**	**Primer sequence**		**Amplicon length**
Fgf4	Fwd	CCGACGAGTGTAAATTCAAAGAAA	97
	Rv	TTCTTACTGAGGGCCATGAACA	
Cilp	Fwd	AAAAAGACGGCTTTCCAAATCA	78
	Rv	GGCATAGATAGGCCCATTGC	
Rxrg	Fwd	CGTTGAGTGGGCCAAACG	75
	Rv	CCTGCCCGGAGTAGAATGAC	
Dll1	Fwd	GACCGCCGCTTCCTAATAGG	74
	Rv	GCCCAGATGTTCAGCTTAATTCC	
Spp1	Fwd	CCCTCGATGTCATCCCTGTT	69
	Rv	TGCCCTTTCCGTTGTTGTC	
Vstm2a	Fwd	GTGGAGCTCTTACCCGACAGA	73
	Rv	CATTGCCTTGGACTTTCACTGTAC	
Figf	Fwd	GGTTGCCTGAAACAGAGTAGTAGGT	71
	Rv	AGCATTGCCCTTGGACTTTG	
Fgf10	Fwd	GGGCTGCTGTTGCTGCTT	94
	Rv	GGCCTCCTGTGACACCATGT	
Sfrp2	Fwd	CAGAGAGAGTTCAAGCGCATCTC	68

### Gene ontology annotation analysis

Gene Ontology (GO) terms were utilised to reveal significant enrichment of groups of genes among the DE datasets from the microarray and the RNA-seq analysis using the Database for Annotation, Visualisation and Integrated Discovery, DAVID (http://david.abcc.ncifcrf.gov/) [28], and GOstat (http://gostat.wehi.edu.au/) software. Analysis of GO terms associated with biological process, molecular function and cellular component was performed on all data-sets independently and combined to identify significantly (p ≤ 0.05) enriched gene sets. The strength of the enrichment of any GO term-associated gene set is reflected in the calculated p-values, comparing the proportion of genes in the data-set and the proportion of genes in the genome bearing that annotation.

### In situ Hybridisation

Expression probes were prepared from cDNA clones obtained from a mouse expressed sequence tag (EST) library (IMAGE, Source Biosciences); details given in Table 8. Antisense and sense digoxigenin-labelled RNA was transcribed *in vitro* from 1 μg of linearised plasmid using T7, T3 and SP6 promoter sites (according to insert orientation and vector), all components for *in vitro* transcription were purchased from Roche, Germany. DNA template was degraded by incubation of probes with RNase free DNase (Roche) and probes purified on G25 columns (Amersham Biosciences, USA) according to manufacturer’s instructions. Probe concentrations were determined by spectophotometry and probes stored at -20°C. Embryonic limbs at TS23 were fixed (4% Paraformaldehyde (PFA)), dehydrated (graded series of Methanol/Phosphate Buffered Saline with 0.1% Triton (PBT) washes) and stored at -20°C prior to sectioning. Limbs were rehydrated in a reverse series of Methanol/PBT washes. Sectioning was performed with a vibrating microtome (VT1000S, Leica; embedded in 4% low melting point agarose (Invitrogen)/PBS (80 μm)). Hybridisation of sections was largely carried out as described previously [128].

**Table 8:** Details of cDNA clones used as expression probes

**Gene**	**Extent of probe on genbank sequence**	**Source**
Sfrp2	Nucleotide 82 to 852 on gene bank sequence U88567	A. Rattner
Cd44	Nucleotide 222 to 3020 on gene bank sequence NM_00103915.1	IMAGE Library
Spp1	Nucleotide 27 to 1472 on gene bank sequence NM_001204201	IMAGE Library
Wnt4	Nucleotide 639 to 1101 on NM_009523.1	A. McMahon

### Availability of supporting data

The data sets supporting the results of this article are available in the EMBL-EBI ArrayExpress repository (http://www.ebi.ac.uk/arrayexpress/). The differential expressed data set from the Microarray [E-MTAB-1744], the differential expressed data set from the RNA-sequencing [E-MTAB-1746] and for the transcriptome [E-MTAB-1745]. Lists of differentially expressed genes are available in Additional file 1.

## Abbreviations

TS: Theiler stage; RNA-sseq: RNA-sequencing; PCR: Polymerase chain reaction; RNA: Ribonucleic acid; RIN: RNA integrity number; cDNA: complementary deoxyribonucleic acid; FDR: False discovery rate; qRT-PCR: quantitative real time PCR; DE: Differential expression; Tm: Melting temperature; bp: Base pairs; ng: Nanogram; μM: Micro molar; min: Minute; Ct: Cycle threshold; GO: Gene ontology; EST: Expressed sequence tag; PFA: Paraformaldehyde; PBS: Phosphate buffer saline.

## Competing interests

The authors declare that they have no competing interests.

## Authors’ contributions

RR carried out all the experiments and analysis of data, collected all samples and drafted the manuscript. NN and PP were involved in preliminary work and conception of the present study. EK, PC and DM provided an RNA sequencing service at TrinSeq generating the primary gene lists. DK contributed to discussions of the findings and drafting of the manuscript. PM conceived and designed the study, participated in data analysis and oversaw drafting of the manuscript. All authors read and approved the final manuscript.

## Supplementary Material

Additional file 1 Supplementary data Tables S1-S4. Click here for file
