# Supplementary material for: Identification of mechanosensitive genes during skeletal development: alteration of genes associated with cytoskeletal rearrangement and cell signalling pathways
Source: BMC Genomics. 2014 Jan 20;15:48. doi: 10.1186/1471-2164-15-48 (PMC3905281; doi:10.1186/1471-2164-15-48)
Supplement: Additional file 1 — Supplementary data Tables S1-S4. [file 1471-2164-15-48-S1.docx]

| Fold change distribution of differentially expressed genes ≥ 2 fold (p-value ≤0.05) from Microarray and RNA-seq analysis. | | | | | | | | | |
| --- | --- | --- | --- | --- | --- | --- | --- | --- | --- |
|  | **Platform** | **Fold Change** | | | | | | | |
|  |  |  | **# Not determined** | **≥100** | **100≤50** | **50≤25** | **25≤10** | **10≤5** | **5≤2** |
| Up-regulated | Microarray |  |  | 0 | 0 | 0 | 0 | 7 | 83 |
|  | RNA-seq |  | 2 | 0 | 0 | 2 | 7 | 35 | 373 |
| Down-regulated | Microarray |  |  | 2 | 16 | 32 | 59 | 67 | 108 |
|  | RNA-seq |  | 23 | 17 | 57 | 59 | 80 | 84 | 298 |
| Total differentially expressed genes | **Microarray** |  |  | **2** | **16** | **32** | **59** | **74** | **192** |
|  | **RNA-seq** |  |  | **42^#^** | **57** | **61** | **87** | **119** | **671** |
| ^#^Not determined as no expression in the control so the precise fold change cannot be assigned  Note that 25 DE genes (2 up-regulated and 23 down-regulated) detected by RNAseq could not be assigned a numerical fold change value because no reads were mapped in one of the sets of samples. The up-regulated genes from both the Microarray and RNA-seq showed a reduced degree of alteration compared to down-regulated genes. In each of the categories between 50 and 5 fold change, there is a similar proportion of total differentially expressed genes with both platforms, however this is disturbed in the lowest fold change category of 5≤2, with the microarray representing 192 genes but the RNA-seq totalling 671. This increase in gene number specifically close to the lower cut off point, again indicates that the RNA-seq platform was more sensitive in identifying significantly DE genes. There is again further support for this if the criteria for selection of differential expression are relaxed for the microarray data; using a corrected p-value of ≤0.08 and fold change ≥ 2, the total number of genes with a fold change 5≤2 increases to 331 from 192 (not shown). | | | | | | | | | |

Supplementary Data Table 1

Supplementary Date Table 2: List of all DE down-regulated genes and fold change estimated by Microarray and RNA-seq.

| **Gene id** | **Mesenchyme enriched ≥3 fold** | **RNA sequencing**  **Down-regulated** | | **Microarray**  **Down-regulated** | |
| --- | --- | --- | --- | --- | --- |
|  |  | **Fold Change** | **p-value** | **Fold Change** | **p-value** |
| Fgf4 |  | #^[[1]](#footnote-1)^ | 0.000638844 | 41.054 | 0.00008 |
| Tmem184a |  | #^1^ | 5.25E-06 | 13.264 | 0.04677 |
| Mep1a |  | #^1^ | 0.010348707 | 10.561 | 0.00645 |
| Dysfip1 |  | #^1^ | 1.33E-07 | 6.812 | 0.01802 |
| Fgf6 | * | #^1^ | 1.11E-11 | 3.175 | 0.04425 |
| 2310050B05Rik |  | #^1^ | 4.13E-06 |  |  |
| 2900079G21Rik |  | #^1^ | 4.76E-07 |  |  |
| Acpt |  | #^1^ | 0.045689548 |  |  |
| Cym |  | #^1^ | 0.041456515 |  |  |
| Foxd4 |  | #^1^ | 1.15E-05 |  |  |
| Havcr1 | * | #^1^ | 5.89E-06 |  |  |
| Lmod2 | * | #^1^ | 2.49E-06 |  |  |
| Mir23b |  | #^1^ | 0.021403052 |  |  |
| Pnmt |  | #^1^ | 0.012794292 |  |  |
| Prame |  | ##^1^ | 1.04E-05 |  |  |
| Ros1 |  | #^1^ | 0.023630339 |  |  |
| Scn10a | * | #^1^ | 0.008723248 |  |  |
| Snai3 |  | #^1^ | 0.000979848 |  |  |
| Stoml3 |  | #^1^ | 0.036230472 |  |  |
| Tekt5 |  | #^1^ | 0.026716473 |  |  |
| Tmsb15a |  | #^1^ | 0.030048338 |  |  |
| Ucp3 | * | #^1^ | 0.031453558 |  |  |
| Yipf7 |  | # | 0.000163307 |  |  |
| A930003A15Rik |  | 231.055 | 0.000287109 | 18.655 | 0.00782 |
| Dbx1 |  | 179.594 | 5.29E-38 | 40.147 | 0.02491 |
| Arx | * | 158.132 | 0.00153276 |  |  |
| Mybph |  | 148.831 | 4.60E-07 | 124.692 | 0.02395 |
| Hspb3 |  | 146.551 | 4.60E-06 |  |  |
| Myf6 | * | 146.233 | 6.78E-06 |  |  |
| Gjd4 |  | 141.023 | 1.48E-05 | 39.542 | 0.00901 |
| Myf5 |  | 131.785 | 7.62E-20 |  |  |
| Adprhl1 |  | 128.451 | 1.68E-06 | 6.578 | 0.01050 |
| Sgcg |  | 125.637 | 3.95E-06 | 4.691 | 0.00645 |
| Sln |  | 115.450 | 2.65E-05 |  |  |
| Chrnd |  | 113.472 | 1.39E-10 |  |  |
| Myh7 |  | 108.322 | 4.98E-11 |  |  |
| Tnnc1 |  | 107.742 | 7.70E-07 | 87.484 | 0.02563 |
| Lbx1 |  | 106.407 | 7.20E-12 | 11.171 | 0.01639 |
| Tmem8c |  | 103.616 | 1.46E-07 | 17.556 | 0.00371 |
| Tceal7 | * | 100.473 | 2.35E-05 |  |  |
| 1110059M19Rik |  | 99.975 | 3.20E-05 |  |  |
| Myoz2 |  | 99.334 | 3.09E-06 |  |  |
| Trim55 | * | 99.055 | 8.55E-08 |  |  |
| Vgll2 |  | 98.294 | 1.39E-13 | 49.049 | 0.01175 |
| Ankrd1 |  | 97.739 | 1.02E-06 | 75.139 | 0.00302 |
| Cox8b |  | 97.681 | 0.001255014 |  |  |
| Csrp3 |  | 96.892 | 1.21E-06 |  |  |
| Actn2 |  | 96.575 | 2.69E-07 |  |  |
| Chrng |  | 96.232 | 9.54E-07 | 12.159 | 0.00627 |
| Popdc3 |  | 94.586 | 0.000172639 | 4.403 | 0.02212 |
| Cacng1 |  | 90.866 | 4.19E-07 | 32.059 | 0.04062 |
| Mstn | * | 90.078 | 4.12E-19 | 50.047 | 0.00709 |
| Hfe2 |  | 87.940 | 2.07E-06 | 50.875 | 0.01598 |
| Smyd1 | * | 87.679 | 3.17E-09 | 13.318 | 0.02829 |
| Myh3 |  | 87.097 | 3.27E-10 |  |  |
| Myl1 |  | 85.959 | 2.39E-05 |  |  |
| Xirp1 | * | 84.349 | 3.06E-05 | 5.065 | 0.00901 |
| Mylpf |  | 82.738 | 5.04E-05 | 26.582 | 0.01289 |
| Actc1 |  | 81.479 | 3.62E-08 |  |  |
| Trim72 |  | 80.325 | 5.71E-11 | 44.156 | 0.02310 |
| Tnnt1 |  | 80.285 | 1.40E-07 | 44.680 | 0.02166 |
| Kbtbd5 |  | 77.398 | 5.15E-08 | 55.169 | 0.02044 |
| Casq2 | * | 76.168 | 7.56E-05 |  |  |
| Lmod3 |  | 75.222 | 6.12E-06 | 32.640 | 0.02212 |
| Tnnc2 |  | 74.725 | 1.91E-06 |  |  |
| Mb |  | 73.707 | 7.16E-06 | 35.531 | 0.01765 |
| Pax7 |  | 73.024 | 2.51E-74 | 23.660 | 0.00901 |
| Ttn | * | 72.937 | 1.86E-08 | 50.449 | 0.00834 |
| Fitm1 |  | 72.173 | 1.34E-09 | 33.058 | 0.03818 |
| Myod1 |  | 71.771 | 5.57E-17 | 45.123 | 0.00670 |
| Tnnt2 |  | 69.284 | 3.10E-06 |  |  |
| Trdn |  | 68.649 | 1.40E-07 | 54.363 | 0.03187 |
| Atp1b4 | * | 67.690 | 1.68E-05 |  |  |
| Best3 | * | 67.237 | 6.34E-09 | 16.822 | 0.00371 |
| Il17b |  | 66.180 | 0.000185033 |  |  |
| Acta1 |  | 65.851 | 9.80E-05 |  |  |
| Mir133b |  | 65.716 | 0.000606002 |  |  |
| Cox6a2 |  | 65.555 | 0.00048648 |  |  |
| Ankrd2 | * | 65.349 | 0.002450587 |  |  |
| Trim54 |  | 64.915 | 5.93E-05 | 33.576 | 0.04568 |
| Krt33b |  | 64.867 | 0.001294308 | 16.038 | 0.01225 |
| Myl2 |  | 64.742 | 0.000253453 | 23.270 | 0.04191 |
| Myom2 |  | 64.557 | 1.96E-09 |  |  |
| Lrrc30 |  | 63.825 | 9.24E-06 |  |  |
| Myh7b |  | 63.715 | 2.77E-11 | 76.442 | 0.04129 |
| 3425401B19Rik |  | 62.341 | 4.16E-06 |  |  |
| Myot | * | 61.078 | 0.000103901 |  |  |
| Kbtbd10 | * | 60.151 | 1.71E-08 | 79.742 | 0.00371 |
| Itgb1bp2 |  | 58.265 | 1.86E-05 |  |  |
| Neb | * | 57.041 | 1.22E-08 | 35.800 | 0.01163 |
| Myl4 |  | 56.963 | 6.14E-05 | 34.577 | 0.03079 |
| Arpp21 |  | 56.688 | 4.24E-11 | 56.372 | 0.00958 |
| Cacna1s |  | 56.678 | 3.78E-07 | 58.404 | 0.03587 |
| Chrna1 |  | 55.033 | 1.37E-09 | 76.917 | 0.00901 |
| Tigd4 |  | 54.824 | 0.000385919 |  |  |
| Myog |  | 53.537 | 2.55E-16 | 51.220 | 0.02105 |
| Atp2a1 |  | 51.426 | 1.36E-06 | 9.285 | 0.00709 |
| Tcap |  | 48.488 | 0.00016188 | 61.371 | 0.01317 |
| Ldb3 | * | 48.169 | 3.41E-05 | 7.006 | 0.03013 |
| C130080G10Rik | * | 48.033 | 0.008148424 |  |  |
| Mybpc1 | * | 47.960 | 5.21E-06 |  |  |
| Alpk3 |  | 47.897 | 2.10E-07 | 42.743 | 0.01598 |
| Myo18b |  | 47.329 | 1.16E-09 | 36.350 | 0.02675 |
| Ampd1 | * | 47.163 | 9.24E-05 |  |  |
| Ripply1 |  | 46.845 | 0.020822695 |  |  |
| Klhl31 | * | 46.120 | 7.67E-06 | 19.278 | 0.02491 |
| 2310002L09Rik | * | 46.103 | 1.07E-07 | 40.300 | 0.02491 |
| Tnni1 |  | 44.819 | 2.11E-05 |  |  |
| Trpv1 |  | 44.032 | 0.00103819 |  |  |
| Atp13a5 |  | 43.397 | 4.89E-06 | 3.964 | 0.03171 |
| Ablim3 |  | 43.325 | 1.57E-08 | 21.901 | 0.01126 |
| Pitx3 |  | 42.860 | 9.67E-14 | 18.771 | 0.01175 |
| Uts2r |  | 42.751 | 9.28E-05 |  |  |
| Ache |  | 42.710 | 4.67E-06 | 27.920 | 0.02629 |
| Gm889 |  | 42.435 | 0.002373688 |  |  |
| Smpx |  | 42.176 | 0.000413451 | 34.338 | 0.00399 |
| Igfbp1 |  | 41.402 | 0.000458505 | 17.520 | 0.00901 |
| Myh8 | * | 40.847 | 4.51E-09 | 19.589 | 0.02510 |
| Tnnt3 |  | 40.508 | 0.00018347 | 17.435 | 0.03668 |
| Rapsn |  | 39.898 | 1.21E-08 |  |  |
| Gm7325 |  | 39.309 | 1.54E-08 | 48.412 | 0.00645 |
| Cyp2ab1 |  | 38.930 | 0.002350375 |  |  |
| Hspb2 |  | 38.350 | 0.000109634 | 22.535 | 0.00627 |
| Srpk3 |  | 37.989 | 0.000171147 | 38.197 | 0.00901 |
| Kremen2 |  | 37.427 | 6.33E-07 | 17.221 | 0.00645 |
| Mypn | * | 36.927 | 4.18E-06 | 14.088 | 0.00327 |
| Coro6 |  | 36.874 | 0.000398936 |  |  |
| Sh3bgr |  | 35.425 | 5.65E-05 | 25.153 | 0.01735 |
| Apobec2 | * | 35.312 | 0.00010877 | 58.828 | 0.03649 |
| Obscn |  | 35.116 | 4.00E-08 | 64.038 | 0.00789 |
| Myl3 |  | 33.794 | 0.003700068 | 27.441 | 0.01610 |
| Cdh15 |  | 32.652 | 1.57E-12 | 22.649 | 0.00575 |
| Abra |  | 31.900 | 0.005512519 |  |  |
| Cacng6 |  | 31.804 | 0.000299637 |  |  |
| Tmem182 | * | 31.716 | 0.004213734 |  |  |
| Synpo2l |  | 31.489 | 1.69E-07 |  |  |
| F2 |  | 31.361 | 0.007704493 | 22.295 | 0.01017 |
| Sypl2 | * | 31.244 | 0.00080006 |  |  |
| Rbm24 |  | 31.077 | 3.57E-10 | 22.326 | 0.00569 |
| Scn3b | * | 30.675 | 3.28E-06 |  |  |
| Cav3 |  | 30.120 | 0.000918982 | 22.934 | 0.00709 |
| Mybpc3 |  | 29.921 | 0.000673869 |  |  |
| Ccnb3 | * | 29.525 | 1.28E-05 |  |  |
| Musk | * | 29.129 | 1.84E-09 | 31.883 | 0.03444 |
| Rgr |  | 28.944 | 0.014988579 |  |  |
| Mylk4 | * | 28.587 | 9.03E-06 | 14.918 | 0.03171 |
| Myom3 |  | 27.767 | 1.08E-07 | 14.330 | 0.03973 |
| Ky |  | 27.403 | 5.08E-07 | 9.045 | 0.02330 |
| Dhrs7c |  | 26.627 | 3.53E-05 | 22.417 | 0.02363 |
| Mlc1 |  | 26.493 | 0.000342179 | 9.746 | 0.02166 |
| Prkag3 |  | 26.251 | 0.000650341 |  |  |
| Tmprss2 |  | 25.890 | 2.74E-05 |  |  |
| Tmod1 |  | 25.608 | 3.13E-06 | 24.435 | 0.03973 |
| Otog |  | 25.430 | 8.59E-09 |  |  |
| Art1 |  | 25.144 | 7.18E-05 | 13.363 | 0.00371 |
| Ckm |  | 25.090 | 0.001359396 |  |  |
| Myoz1 |  | 24.844 | 0.000350615 | 18.794 | 0.02491 |
| Mylk2 |  | 24.679 | 0.00136607 | 2.852 | 0.04700 |
| Alpk2 | * | 24.629 | 7.57E-09 | 5.427 | 0.01050 |
| Itgb6 | * | 24.278 | 3.85E-06 | 27.782 | 0.01388 |
| Crhr2 | * | 24.268 | 0.004984818 |  |  |
| Jsrp1 |  | 22.778 | 0.000314804 | 10.029 | 0.02491 |
| Tnni2 |  | 22.733 | 0.000708664 | 14.431 | 0.03055 |
| Cblc |  | 22.719 | 0.046710202 |  |  |
| Ptx4 |  | 21.644 | 2.31E-17 |  |  |
| Xirp2 | * | 20.984 | 3.73E-06 |  |  |
| Lrtm1 | * | 20.551 | 0.000894063 |  |  |
| Msc |  | 20.402 | 3.53E-22 | 15.969 | 0.02491 |
| Stac3 |  | 20.398 | 5.43E-06 | 9.553 | 0.04044 |
| Btbd17 |  | 20.223 | 7.70E-11 | 18.216 | 0.00738 |
| Sgca |  | 19.899 | 6.32E-05 | 13.827 | 0.01633 |
| Grip2 |  | 19.683 | 0.000641698 |  |  |
| Dok7 |  | 19.673 | 1.98E-10 | 15.493 | 0.01958 |
| Klrb1c |  | 19.361 | 0.034210789 |  |  |
| Fhad1 |  | 19.117 | 0.000146456 | 13.893 | 0.02110 |
| Wdr16 |  | 18.658 | 0.020618305 | 35.858 | 0.02113 |
| Barx2 |  | 18.601 | 5.29E-23 |  |  |
| Ckmt2 |  | 18.480 | 0.009086687 |  |  |
| Des |  | 18.470 | 3.71E-05 | 14.972 | 0.00901 |
| Mir133a-2 |  | 18.359 | 0.026213942 |  |  |
| Marveld2 |  | 18.354 | 8.73E-07 | 9.939 | 0.02491 |
| Elovl3 |  | 18.044 | 4.46E-09 |  |  |
| Slc44a3 | * | 17.993 | 9.50E-07 |  |  |
| Murc | * | 17.703 | 0.000725054 | 15.510 | 0.02212 |
| Krt4 | * | 17.664 | 0.010105871 |  |  |
| Ryr1 |  | 17.564 | 6.05E-11 | 11.484 | 0.02110 |
| Unc45b |  | 17.298 | 4.20E-05 | 7.124 | 0.02110 |
| Rtn2 |  | 16.606 | 3.50E-05 | 14.053 | 0.00627 |
| Ccin | * | 16.535 | 0.04105955 |  |  |
| 1500017E21Rik |  | 16.501 | 4.58E-07 |  |  |
| Zfp474 |  | 16.477 | 0.00741475 |  |  |
| Gm5105 | * | 16.465 | 2.29E-07 |  |  |
| Fam159a |  | 15.637 | 0.000139788 | 6.503 | 0.02207 |
| Frmpd1 |  | 15.590 | 3.25E-19 | 9.015 | 0.00627 |
| Pgam2 |  | 15.539 | 0.00097618 | 19.873 | 0.01050 |
| Asb2 |  | 15.411 | 0.00025413 | 9.920 | 0.00374 |
| Duoxa1 |  | 15.409 | 0.001254674 | 4.989 | 0.03018 |
| Casz1 |  | 15.171 | 1.55E-09 | 25.350 | 0.02291 |
| Gja3 | * | 15.158 | 0.031671581 |  |  |
| Myom1 |  | 15.117 | 3.75E-05 | 12.371 | 0.02290 |
| Myl6b |  | 14.795 | 0.000124537 | 11.872 | 0.00503 |
| Kcnc4 |  | 14.708 | 0.003835808 |  |  |
| Klhl38 |  | 14.705 | 6.02E-06 |  |  |
| Hhatl |  | 14.547 | 0.001515536 | 4.586 | 0.03107 |
| Popdc2 |  | 14.543 | 1.01E-07 |  |  |
| Colq | * | 13.793 | 0.000636245 | 6.168 | 0.04554 |
| Kcnk13 | * | 13.454 | 1.28E-06 |  |  |
| Tpm2 |  | 13.435 | 0.000344311 | 2.302 | 0.04191 |
| Nrap |  | 13.367 | 0.000125314 |  |  |
| Chrnb1 |  | 13.347 | 1.34E-05 | 7.600 | 0.01831 |
| Myh6 | * | 13.294 | 0.000129867 | 8.711 | 0.01765 |
| Slc25a18 |  | 13.092 | 0.000244044 |  |  |
| P2rx5 |  | 12.746 | 3.43E-05 |  |  |
| Capn3 |  | 12.740 | 0.000703589 | 8.047 | 0.01184 |
| Gm572 |  | 12.481 | 0.030996655 |  |  |
| Rai2 |  | 12.397 | 0.000190941 | 8.946 | 0.00709 |
| Rxrg |  | 12.290 | 3.24E-05 | 7.048 | 0.00645 |
| Scn4a |  | 12.018 | 1.14E-11 |  |  |
| Fsd2 | * | 11.845 | 0.005482517 |  |  |
| Gal3st2 |  | 11.565 | 0.029992903 |  |  |
| Sag |  | 11.513 | 4.76E-05 |  |  |
| Trim63 |  | 11.172 | 0.000444857 |  |  |
| Arhgap36 | * | 11.080 | 0.007757438 |  |  |
| Impg1 |  | 11.069 | 0.015160443 |  |  |
| Ect2l |  | 10.884 | 0.030198614 |  |  |
| Cck |  | 10.841 | 0.00024209 | 6.888 | 0.02336 |
| Mustn1 |  | 10.824 | 7.41E-05 | 7.415 | 0.00682 |
| Srl | * | 10.785 | 0.000288666 | 12.709 | 0.02491 |
| Tppp3 |  | 10.647 | 1.63E-10 | 9.028 | 0.01050 |
| Stmn4 |  | 10.587 | 5.69E-17 | 8.410 | 0.03036 |
| Thbs4 |  | 10.579 | 1.25E-57 |  |  |
| Gjd2 |  | 10.558 | 0.007216651 |  |  |
| Kbtbd12 | * | 10.438 | 0.013183456 | 16.975 | 0.02743 |
| Sct |  | 10.260 | 0.000351532 | 5.540 | 0.03042 |
| 1600029D21Rik |  | 10.104 | 0.0078423 | 5.124 | 0.01578 |
| Slc6a13 |  | 10.021 | 0.004168164 |  |  |
| Adam2 |  | 9.895 | 0.0211861 |  |  |
| Cilp |  | 9.760 | 5.73E-25 | 9.731 | 0.00645 |
| L3mbtl |  | 9.449 | 0.032843435 |  |  |
| Pygm |  | 9.409 | 0.001093758 | 4.666 | 0.01341 |
| Hspb1 |  | 9.199 | 0.001579204 | 6.302 | 0.01307 |
| Fbxl22 |  | 9.195 | 0.000212027 | 13.370 | 0.00740 |
| Mir3072 |  | 9.024 | 0.003198372 |  |  |
| Akap6 | * | 8.846 | 0.000236632 |  |  |
| Pdlim3 |  | 8.838 | 0.002436503 | 5.509 | 0.01175 |
| Odf3l1 |  | 8.787 | 0.001335064 |  |  |
| Snora43 |  | 8.705 | 0.046050196 |  |  |
| Myo5b | * | 8.630 | 0.005148846 | 10.057 | 0.01194 |
| Ntrk1 |  | 8.503 | 6.65E-05 |  |  |
| Ctnna3 | * | 8.473 | 0.000724585 |  |  |
| Folr4 |  | 8.420 | 0.000211253 |  |  |
| Traf3ip3 |  | 8.344 | 0.003080134 | 4.674 | 0.00881 |
| Gnb3 |  | 8.324 | 0.039793053 |  |  |
| Cryab |  | 8.192 | 0.000348192 | 5.632 | 0.00649 |
| Txlnb | * | 8.106 | 0.000781947 | 7.191 | 0.03042 |
| Kcnc1 | * | 8.064 | 5.02E-06 |  |  |
| Slc28a2 |  | 7.901 | 2.56E-05 | 5.266 | 0.02310 |
| Spink5 |  | 7.880 | 0.000118872 | 6.518 | 0.04425 |
| Rbm20 |  | 7.791 | 4.89E-34 |  |  |
| Usp44 |  | 7.765 | 0.002121462 |  |  |
| Crym |  | 7.748 | 1.05E-05 | 5.159 | 0.02207 |
| Ablim2 |  | 7.636 | 0.005358528 |  |  |
| Frem2 | * | 7.520 | 2.27E-05 | 5.203 | 0.00901 |
| Calb2 |  | 7.442 | 0.00381292 |  |  |
| Rrad |  | 7.381 | 0.001844232 | 5.083 | 0.00371 |
| Art5 |  | 7.313 | 0.001876818 | 6.279 | 0.02675 |
| Mt3 |  | 7.302 | 0.00142808 |  |  |
| Hspb7 |  | 7.054 | 0.04163647 |  |  |
| Ppp1r3a | * | 6.978 | 0.03150198 |  |  |
| Synpo2 | * | 6.952 | 7.65E-06 |  |  |
| Iqsec3 |  | 6.849 | 2.06E-08 | 13.668 | 0.03218 |
| Etv4 |  | 6.796 | 4.50E-13 |  |  |
| Shc3 |  | 6.735 | 0.008690025 |  |  |
| Dusp13 |  | 6.642 | 0.03234326 | 18.884 | 0.00894 |
| Tceal6 |  | 6.638 | 0.010139096 |  |  |
| Gatm | * | 6.550 | 2.53E-05 |  |  |
| Ufsp1 |  | 6.540 | 0.000987288 | 3.081 | 0.02563 |
| Amtn |  | 6.450 | 6.37E-17 |  |  |
| Dusp27 |  | 6.311 | 0.001475933 | 3.935 | 0.01122 |
| LOC100302688 |  | 6.256 | 0.006530937 |  |  |
| Klhl30 |  | 6.212 | 0.003567934 | 5.012 | 0.01694 |
| Rbfox1 |  | 6.190 | 0.004453688 |  |  |
| Gpr83 |  | 6.152 | 3.21E-12 |  |  |
| Kcnj11 |  | 6.133 | 0.020393335 |  |  |
| Tecrl | * | 6.093 | 1.22E-05 | 5.844 | 0.02675 |
| Slc18a1 |  | 6.060 | 2.06E-05 | 4.951 | 0.03669 |
| Acta2 |  | 6.045 | 0.00027772 | 4.818 | 0.04956 |
| Enkur |  | 5.975 | 0.000384266 |  |  |
| Ank1 |  | 5.965 | 6.86E-07 | 12.518 | 0.00371 |
| Islr2 |  | 5.955 | 6.65E-11 | 5.198 | 0.00901 |
| Mir25 |  | 5.944 | 0.041998686 |  |  |
| Atp1a2 |  | 5.864 | 0.001075117 |  |  |
| Htr2b | * | 5.853 | 0.030107151 | 12.825 | 0.00901 |
| Cpa5 |  | 5.778 | 5.29E-06 |  |  |
| Cecr2 |  | 5.750 | 6.03E-12 |  |  |
| LOC100499420 |  | 5.739 | 0.00032585 |  |  |
| Dnahc5 |  | 5.641 | 0.000310299 |  |  |
| Dner |  | 5.639 | 5.36E-07 |  |  |
| Car3 | * | 5.611 | 0.000596286 |  |  |
| Ramp1 |  | 5.525 | 0.040157864 |  |  |
| Capn11 |  | 5.519 | 0.004820907 |  |  |
| Tslp |  | 5.485 | 3.92E-06 | 3.504 | 0.02683 |
| Serpina3n |  | 5.472 | 0.002075378 | 6.027 | 0.03903 |
| Nes |  | 5.461 | 2.95E-10 | 5.573 | 0.00371 |
| Ovgp1 |  | 5.449 | 0.006627217 |  |  |
| Sync |  | 5.425 | 5.86E-10 | 5.719 | 0.03516 |
| AF366264 |  | 5.396 | 0.042800741 |  |  |
| 2310007A19Rik |  | 5.350 | 0.003881734 |  |  |
| Slc2a5 |  | 5.271 | 5.39E-08 |  |  |
| Fgf8 |  | 5.254 | 0.015882792 |  |  |
| Tuba4a |  | 5.238 | 0.004065209 |  |  |
| Smtnl2 |  | 5.180 | 9.01E-08 | 4.730 | 0.04271 |
| Nol3 |  | 5.179 | 0.00243595 | 3.481 | 0.02908 |
| Clcnka |  | 5.177 | 0.007705476 |  |  |
| Adssl1 |  | 5.123 | 0.022447771 | 4.378 | 0.00901 |
| F2rl1 |  | 5.106 | 3.92E-18 | 3.050 | 0.04447 |
| ORF63 |  | 5.102 | 0.006766012 |  |  |
| Fgf5 | * | 5.098 | 4.04E-05 |  |  |
| Cdk5r1 |  | 5.062 | 3.26E-18 | 3.930 | 0.00901 |
| Bex1 |  | 5.060 | 0.003064203 | 2.285 | 0.02491 |
| Ostn |  | 4.996 | 0.032133165 |  |  |
| Tspan33 |  | 4.983 | 8.36E-06 | 3.308 | 0.02310 |
| Rimbp3 |  | 4.972 | 0.020203059 |  |  |
| Adora1 |  | 4.950 | 0.001180885 |  |  |
| Zmynd17 |  | 4.939 | 0.000574852 |  |  |
| Cap2 | * | 4.937 | 0.000200587 | 4.186 | 0.04786 |
| Aifm3 |  | 4.912 | 0.000929811 |  |  |
| Pitx2 |  | 4.874 | 0.000814308 |  |  |
| Rasl10a |  | 4.821 | 0.003305427 | 4.124 | 0.01373 |
| Cd3g |  | 4.749 | 0.041723914 |  |  |
| Hpcal4 |  | 4.745 | 0.026598756 |  |  |
| Casq1 |  | 4.735 | 0.033530375 | 3.068 | 0.01050 |
| Tas1r1 |  | 4.695 | 0.043219376 | 6.324 | 0.01901 |
| Gm11346 |  | 4.624 | 0.037549509 |  |  |
| Rprm |  | 4.615 | 0.014824336 |  |  |
| Rit2 |  | 4.607 | 7.36E-06 |  |  |
| 4933436C20Rik |  | 4.591 | 0.000815015 |  |  |
| Snord99 |  | 4.571 | 0.001661383 |  |  |
| Fgfr4 |  | 4.545 | 6.99E-23 | 3.231 | 0.00933 |
| Sh2b2 |  | 4.525 | 6.59E-05 | 2.988 | 0.04328 |
| Spon2 |  | 4.490 | 1.29E-09 | 5.173 | 0.00627 |
| Fam78a |  | 4.482 | 0.001494111 | 3.724 | 0.00560 |
| D930048N14Rik |  | 4.426 | 0.009625517 |  |  |
| Snord83b |  | 4.424 | 0.046506713 |  |  |
| Macrod1 |  | 4.419 | 0.002397058 |  |  |
| Tnfsf13b |  | 4.412 | 0.045710756 |  |  |
| Ppp1r14c | * | 4.402 | 0.004896588 |  |  |
| Ddc |  | 4.399 | 0.000150515 | 2.720 | 0.03347 |
| Tmem88b |  | 4.362 | 4.89E-10 |  |  |
| Slc30a3 |  | 4.350 | 0.008431678 |  |  |
| Adcyap1r1 |  | 4.332 | 9.57E-17 | 3.919 | 0.02974 |
| Kcne1l |  | 4.331 | 2.28E-06 |  |  |
| 4932438H23Rik | * | 4.324 | 0.045421555 |  |  |
| Rgs16 |  | 4.318 | 4.08E-11 |  |  |
| 5930412G12Rik | * | 4.264 | 0.011044667 |  |  |
| Gpr120 |  | 4.171 | 0.035698806 |  |  |
| Jph2 |  | 4.160 | 0.000156224 |  |  |
| Tac1 |  | 4.139 | 2.18E-05 |  |  |
| Sept04 |  | 4.087 | 1.98E-06 |  |  |
| A430110N23Rik |  | 4.081 | 2.89E-27 |  |  |
| Tex14 |  | 4.070 | 0.000570692 | 5.613 | 0.02023 |
| Rragd |  | 4.052 | 2.17E-08 |  |  |
| 1700113I22Rik |  | 4.024 | 0.000731808 | 2.601 | 0.00709 |
| Htra4 |  | 4.015 | 7.54E-07 |  |  |
| Slc35f3 |  | 4.014 | 2.10E-08 | 3.144 | 0.03983 |
| Megf10 |  | 4.009 | 1.37E-24 | 3.488 | 0.02491 |
| Esrrb |  | 3.990 | 0.000491894 | 3.647 | 0.01598 |
| Zfp352 |  | 3.936 | 0.012205983 |  |  |
| Fndc5 |  | 3.925 | 0.00050277 |  |  |
| Gm1574 |  | 3.920 | 0.002391272 | 6.132 | 0.02675 |
| Pabpc1l |  | 3.898 | 0.001062351 |  |  |
| Insrr |  | 3.891 | 0.000788901 |  |  |
| Dmpk |  | 3.877 | 0.00326029 | 2.705 | 0.02212 |
| Tead4 | * | 3.860 | 3.03E-13 | 4.381 | 0.03649 |
| Myo16 |  | 3.848 | 3.94E-08 |  |  |
| Iffo1 |  | 3.837 | 0.000289585 | 2.640 | 0.01289 |
| BY080835 |  | 3.826 | 0.016938161 |  |  |
| Cnr1 |  | 3.818 | 1.52E-13 | 2.590 | 0.00399 |
| Tll2 | * | 3.805 | 0.000121063 |  |  |
| Mirg |  | 3.791 | 0.016388196 |  |  |
| Sema6b |  | 3.779 | 1.95E-07 |  |  |
| Camk2a |  | 3.776 | 0.000910514 |  |  |
| Ppfia4 |  | 3.766 | 0.005944714 |  |  |
| Camk2b |  | 3.762 | 0.000587067 |  |  |
| Pou3f1 |  | 3.761 | 7.85E-05 |  |  |
| Fabp3 |  | 3.748 | 0.046045376 | 2.962 | 0.04425 |
| Vgll3 | * | 3.743 | 7.18E-20 |  |  |
| Trp63 | * | 3.724 | 0.024720208 |  |  |
| Col19a1 | * | 3.629 | 6.57E-05 |  |  |
| Snord65 |  | 3.628 | 0.049056944 |  |  |
| 6230400D17Rik |  | 3.615 | 0.023809995 |  |  |
| Dok5 |  | 3.607 | 0.021244085 |  |  |
| Tmem38a |  | 3.593 | 0.018014843 |  |  |
| C4b |  | 3.591 | 0.002928394 | 4.261 | 0.02829 |
| Fzd10 | * | 3.577 | 0.021714725 |  |  |
| Gpr37 |  | 3.571 | 0.00851303 | 3.684 | 0.02983 |
| Jakmip3 |  | 3.569 | 0.023481256 |  |  |
| 6430562O15Rik |  | 3.499 | 0.005665791 |  |  |
| Spnb1 | * | 3.493 | 0.00122522 |  |  |
| Lrrc39 |  | 3.473 | 0.018679961 |  |  |
| Erbb3 |  | 3.457 | 1.10E-16 |  |  |
| Cobl |  | 3.448 | 0.010742417 | 20.443 | 0.03640 |
| Coch |  | 3.419 | 4.76E-07 | 3.814 | 0.03461 |
| 2900005J15Rik |  | 3.402 | 0.029056216 |  |  |
| Bcam |  | 3.389 | 0.00124051 |  |  |
| Prkcc |  | 3.371 | 0.018418282 |  |  |
| Hrc |  | 3.360 | 0.004990099 | 3.130 | 0.04550 |
| Atp2b3 | * | 3.356 | 0.016922185 |  |  |
| Arg1 |  | 3.355 | 0.017844783 |  |  |
| Cdkn1a |  | 3.317 | 0.000390277 |  |  |
| Tnfrsf12a |  | 3.305 | 4.85E-05 |  |  |
| Ntn5 |  | 3.299 | 0.003150271 |  |  |
| Chodl |  | 3.292 | 1.38E-05 | 3.152 | 0.04328 |
| Zfp238 |  | 3.284 | 4.78E-08 |  |  |
| Gldc |  | 3.263 | 0.000326193 |  |  |
| Tmem116 |  | 3.253 | 0.017991955 |  |  |
| Dpf3 |  | 3.251 | 0.032786354 |  |  |
| Spry1 |  | 3.243 | 2.75E-18 |  |  |
| Spry4 |  | 3.219 | 3.86E-18 |  |  |
| Gmpr |  | 3.219 | 0.026099494 | 2.018 | 0.02337 |
| Trim17 |  | 3.216 | 0.04712073 |  |  |
| Ccdc141 | * | 3.212 | 0.000148407 |  |  |
| Cnnm1 |  | 3.211 | 7.19E-06 |  |  |
| Myh1 | * | 3.185 | 0.013884554 |  |  |
| D17H6S56E-3 |  | 3.172 | 0.032055615 |  |  |
| Mip |  | 3.168 | 0.013720797 |  |  |
| Dll1 |  | 3.162 | 1.73E-06 | 2.210 | 0.03516 |
| Snap91 |  | 3.162 | 0.007990301 |  |  |
| Mir17hg |  | 3.161 | 5.44E-05 |  |  |
| Meg3 |  | 3.155 | 0.030983905 |  |  |
| Jam2 |  | 3.148 | 0.036344194 | 3.594 | 0.03717 |
| Unc5a |  | 3.138 | 0.004421143 |  |  |
| Plch1 |  | 3.137 | 1.23E-05 | 4.047 | 0.00920 |
| Tnmd |  | 3.134 | 6.59E-13 |  |  |
| 6330403A02Rik |  | 3.126 | 0.000159805 |  |  |
| Gprc5a |  | 3.117 | 8.03E-05 |  |  |
| Lhfpl1 |  | 3.116 | 4.10E-08 |  |  |
| Rbm38 |  | 3.116 | 0.012501553 |  |  |
| C1ql3 |  | 3.106 | 2.75E-08 |  |  |
| Cited1 |  | 3.103 | 0.000277615 |  |  |
| Hspb8 |  | 3.095 | 0.002956441 |  |  |
| Prg4 |  | 3.046 | 1.52E-07 |  |  |
| Snora34 |  | 3.038 | 0.049099265 |  |  |
| Snord52 |  | 3.010 | 0.011579628 |  |  |
| Nup210 |  | 3.006 | 1.07E-14 | 3.294 | 0.03042 |
| Cenpv |  | 2.996 | 8.48E-06 |  |  |
| Ambn |  | 2.965 | 0.00160442 |  |  |
| Snora16a |  | 2.962 | 0.047384799 |  |  |
| Dleu7 |  | 2.962 | 0.017802886 |  |  |
| Kirrel2 |  | 2.960 | 0.047889124 |  |  |
| Gpr20 |  | 2.955 | 8.98E-05 |  |  |
| Itga4 | * | 2.948 | 9.97E-11 | 3.381 | 0.03359 |
| Pde1c |  | 2.946 | 3.42E-08 |  |  |
| B3galt2 | * | 2.923 | 0.036346769 |  |  |
| Snhg7 |  | 2.919 | 0.006088416 |  |  |
| Palmd |  | 2.902 | 0.023169218 |  |  |
| Spp1 |  | 2.901 | 0.043857363 |  |  |
| Doc2g |  | 2.898 | 0.04842582 |  |  |
| Gcom1 |  | 2.896 | 0.005646488 |  |  |
| Itga7 |  | 2.849 | 0.000684807 |  |  |
| Pdlim1 |  | 2.848 | 1.45E-11 |  |  |
| 1810041L15Rik | * | 2.820 | 0.002356183 | 2.965 | 0.00901 |
| Lrrn1 | * | 2.813 | 0.003220508 | 2.566 | 0.03397 |
| Meox1 |  | 2.796 | 2.27E-11 |  |  |
| Kcnj10 |  | 2.792 | 0.02273419 |  |  |
| Mfsd2a |  | 2.791 | 9.64E-06 |  |  |
| Tm6sf1 |  | 2.783 | 0.023438649 | 2.571 | 0.01333 |
| Bin1 |  | 2.782 | 0.000548241 | 2.032 | 0.04754 |
| Prox1 | * | 2.778 | 1.75E-05 |  |  |
| Pknox2 |  | 2.773 | 1.18E-08 |  |  |
| Spry2 |  | 2.755 | 6.11E-14 |  |  |
| Zic3 |  | 2.745 | 0.004207025 |  |  |
| Frmd3 |  | 2.745 | 2.09E-05 |  |  |
| Hbegf |  | 2.741 | 0.000350767 |  |  |
| Ankrd23 |  | 2.736 | 0.021544801 |  |  |
| Shroom3 |  | 2.713 | 0.002091898 |  |  |
| Tmem100 |  | 2.707 | 5.15E-10 |  |  |
| Tubb6 |  | 2.693 | 0.001035104 |  |  |
| Slco5a1 | * | 2.691 | 0.004377646 |  |  |
| Eno3 | * | 2.687 | 0.034131718 |  |  |
| Hes6 |  | 2.686 | 0.001946887 |  |  |
| Col22a1 |  | 2.680 | 0.011807048 |  |  |
| Dynlt1a |  | 2.675 | 0.036885253 |  |  |
| Nexn |  | 2.652 | 0.014639095 |  |  |
| Gm14492 |  | 2.634 | 0.011746406 |  |  |
| Angptl7 |  | 2.632 | 0.012651786 |  |  |
| Ehbp1l1 |  | 2.631 | 0.00150257 |  |  |
| Sh2d5 |  | 2.616 | 0.044303872 |  |  |
| Arhgdig |  | 2.616 | 0.000649376 |  |  |
| Rb1 | * | 2.614 | 0.000176926 | 2.391 | 0.00715 |
| 4933403O08Rik | * | 2.604 | 0.016456786 |  |  |
| 2010204K13Rik |  | 2.599 | 1.45E-05 |  |  |
| Aknad1 | * | 2.590 | 0.003212914 |  |  |
| Asb10 |  | 2.578 | 0.006471367 |  |  |
| Il20rb |  | 2.578 | 0.002114249 |  |  |
| 2210403K04Rik |  | 2.577 | 0.011615356 |  |  |
| Nptx1 |  | 2.577 | 0.000390855 |  |  |
| Dok3 |  | 2.569 | 0.049938133 |  |  |
| Lgi1 |  | 2.565 | 0.002611874 |  |  |
| Vamp1 |  | 2.557 | 0.038815305 |  |  |
| Glipr2 | * | 2.554 | 3.12E-13 |  |  |
| Sim1 | * | 2.553 | 0.033426892 |  |  |
| P2rx6 |  | 2.550 | 0.035400757 | 9.052 | 0.03973 |
| Nnmt |  | 2.549 | 0.005961276 | 2.407 | 0.04361 |
| Dtx4 |  | 2.544 | 3.50E-12 |  |  |
| Camkv |  | 2.540 | 0.003666581 |  |  |
| Tdrd1 |  | 2.534 | 1.58E-05 |  |  |
| Tnxb |  | 2.528 | 1.10E-06 |  |  |
| Ppp1r14b |  | 2.526 | 0.000513015 |  |  |
| Tchh |  | 2.525 | 0.032617528 |  |  |
| Sall1 |  | 2.519 | 0.009263167 |  |  |
| Tbc1d8 |  | 2.515 | 1.81E-05 |  |  |
| Chrm3 |  | 2.512 | 0.000313547 |  |  |
| Prkcq |  | 2.509 | 1.99E-06 | 2.254 | 0.01637 |
| Plekhh1 |  | 2.500 | 3.68E-05 | 3.040 | 0.02140 |
| Ank2 |  | 2.495 | 3.07E-13 |  |  |
| Mir3064 |  | 2.484 | 0.031260542 |  |  |
| Hsd11b1 |  | 2.483 | 0.009085944 |  |  |
| Cidea |  | 2.475 | 0.016861321 |  |  |
| Tnik | * | 2.470 | 3.19E-09 |  |  |
| E2f2 |  | 2.460 | 1.93E-10 |  |  |
| Atcay |  | 2.457 | 0.018944588 |  |  |
| Aldh1a3 |  | 2.451 | 2.38E-08 |  |  |
| Pdlim4 |  | 2.449 | 0.000822125 |  |  |
| Scx |  | 2.444 | 0.002779351 |  |  |
| Ngfr |  | 2.444 | 2.43E-08 |  |  |
| Bves |  | 2.436 | 0.018857391 |  |  |
| Col20a1 |  | 2.432 | 1.37E-06 |  |  |
| E330033B04Rik |  | 2.427 | 0.039694776 |  |  |
| Ankrd33b |  | 2.419 | 0.03187479 |  |  |
| Gamt |  | 2.411 | 0.032919827 |  |  |
| Tmem150c |  | 2.410 | 0.000118831 |  |  |
| Pip5k1b |  | 2.406 | 0.003669955 |  |  |
| Ttyh2 |  | 2.403 | 2.55E-10 | 2.039 | 0.02337 |
| Ak1 |  | 2.391 | 0.047782606 |  |  |
| Tppp |  | 2.385 | 5.99E-05 |  |  |
| Tceal5 |  | 2.360 | 0.002343191 |  |  |
| Tubb2b |  | 2.358 | 7.40E-08 |  |  |
| Ckb |  | 2.354 | 8.98E-06 |  |  |
| Spg21 |  | 2.339 | 1.17E-10 |  |  |
| Gja5 |  | 2.332 | 0.007499588 |  |  |
| 5330417C22Rik |  | 2.332 | 0.041260172 | 9.153 | 0.02793 |
| Dpep1 |  | 2.330 | 0.03652931 |  |  |
| Tcf15 |  | 2.324 | 0.000893465 |  |  |
| Adrb2 |  | 2.319 | 0.00525532 |  |  |
| Met |  | 2.317 | 1.49E-08 |  |  |
| Hap1 |  | 2.305 | 0.00247612 |  |  |
| Mycl1 |  | 2.300 | 2.17E-07 |  |  |
| Dpysl5 |  | 2.298 | 7.19E-07 |  |  |
| Anxa8 |  | 2.298 | 0.013554134 |  |  |
| Stk32a |  | 2.280 | 0.018216663 |  |  |
| Pla2g4c |  | 2.279 | 0.023239176 |  |  |
| Csdc2 |  | 2.273 | 0.036761566 |  |  |
| Mir351 |  | 2.268 | 0.029594667 |  |  |
| C2cd4c |  | 2.267 | 0.040396409 |  |  |
| En1 |  | 2.267 | 0.004580757 |  |  |
| Flnc |  | 2.256 | 0.000125003 | 2.556 | 0.00645 |
| Pdgfa |  | 2.250 | 5.24E-08 | 2.044 | 0.03998 |
| Shb |  | 2.248 | 0.000601169 |  |  |
| Il1f9 |  | 2.247 | 0.004353474 |  |  |
| Nxph3 |  | 2.244 | 0.044254319 |  |  |
| Il33 |  | 2.234 | 1.77E-05 |  |  |
| Arc |  | 2.221 | 0.027556014 |  |  |
| 2700023E23Rik |  | 2.211 | 0.00489536 |  |  |
| Atf3 |  | 2.211 | 7.60E-05 |  |  |
| Lamb3 |  | 2.210 | 0.001980772 | 3.323 | 0.03781 |
| Padi2 | * | 2.210 | 0.018492502 | 2.303 | 0.02337 |
| Creld1 |  | 2.208 | 0.009965288 |  |  |
| Kcnip4 |  | 2.204 | 0.000764541 |  |  |
| Rtl1 |  | 2.204 | 0.023526084 |  |  |
| Slc29a4 |  | 2.202 | 0.002415868 |  |  |
| Dusp6 |  | 2.197 | 4.43E-09 |  |  |
| Pls1 |  | 2.190 | 0.012618883 |  |  |
| Prdm8 |  | 2.186 | 0.016649886 |  |  |
| Cd44 |  | 2.183 | 1.40E-05 | 2.280 | 0.03042 |
| Epcam | * | 2.178 | 0.005086962 |  |  |
| Kera |  | 2.173 | 0.000317803 |  |  |
| Cfl2 |  | 2.173 | 0.001038403 |  |  |
| Mical2 |  | 2.171 | 3.58E-07 | 4.486 | 0.01225 |
| Rassf5 |  | 2.170 | 0.001317556 |  |  |
| Dusp14 |  | 2.166 | 0.000189953 |  |  |
| Egr2 |  | 2.165 | 0.000831611 |  |  |
| Pvrl1 |  | 2.165 | 3.22E-05 |  |  |
| Akap5 |  | 2.162 | 0.000550736 |  |  |
| Tbx1 |  | 2.160 | 0.046476272 | 2.509 | 0.04139 |
| Rab40b |  | 2.156 | 0.000857314 |  |  |
| Arhgap26 |  | 2.152 | 5.41E-07 | 2.049 | 0.04587 |
| Magel2 |  | 2.148 | 0.000186304 |  |  |
| Pkia | * | 2.142 | 0.001160308 |  |  |
| Snord47 |  | 2.140 | 0.008321211 |  |  |
| 6530418L21Rik |  | 2.123 | 3.70E-07 | 2.191 | 0.02113 |
| Lfng |  | 2.120 | 7.95E-06 |  |  |
| Dbndd1 |  | 2.119 | 0.011743153 |  |  |
| Adam1a |  | 2.118 | 0.021037005 |  |  |
| Ccdc134 |  | 2.113 | 0.003468266 |  |  |
| 1700094D03Rik |  | 2.109 | 0.01745263 |  |  |
| Tmem108 |  | 2.096 | 0.044938087 |  |  |
| Sik1 |  | 2.093 | 0.004105596 |  |  |
| Jag2 |  | 2.087 | 0.002065917 |  |  |
| Htra3 |  | 2.077 | 6.80E-09 |  |  |
| Dnaic2 |  | 2.076 | 0.049783398 |  |  |
| Afap1l1 |  | 2.046 | 0.000727837 |  |  |
| Qprt |  | 2.041 | 0.008007932 |  |  |
| Hn1 |  | 2.036 | 0.000326606 |  |  |
| Sod3 |  | 2.036 | 0.045428703 |  |  |
| Chrna4 |  | 2.035 | 5.32E-05 |  |  |
| Aoc2 |  | 2.034 | 0.014517152 |  |  |
| Chd7 |  | 2.029 | 5.11E-07 |  |  |
| Shisa2 |  | 2.029 | 3.39E-08 |  |  |
| Adamts20 |  | 2.028 | 8.50E-05 |  |  |
| Spink2 |  | 2.021 | 0.0189441 |  |  |
| Cdh4 |  | 2.014 | 1.27E-06 | 2.007 | 0.03107 |
| Plat |  | 2.013 | 7.79E-05 |  |  |
| Arid5a |  | 2.009 | 0.002451172 | 2.060 | 0.03347 |
| Zfp185 |  | 2.008 | 2.97E-08 |  |  |
| AW551984 | * | 2.003 | 0.047744733 |  |  |
| Rasef |  | 2.003 | 0.027683519 |  |  |
| Gls2 |  | 2.000 | 0.010016291 |  |  |
| 4930512H18Rik |  |  |  | 65.074 | 0.02110 |
| LOC100044755 |  |  |  | 38.678 | 0.04644 |
| Myadml2 | * |  |  | 38.177 | 0.00645 |
| 1110002E22Rik |  |  |  | 35.846 | 0.02491 |
| 2310015B20Rik |  |  |  | 28.113 | 0.00627 |
| Dmd | * |  |  | 18.652 | 0.03538 |
| Itgb1bp3 | * |  |  | 11.834 | 0.02928 |
| Klk1b26 |  |  |  | 11.326 | 0.00627 |
| Gm6880 |  |  |  | 9.406 | 0.01815 |
| Gm2252 |  |  |  | 9.359 | 0.04924 |
| Tpm3 |  |  |  | 8.967 | 0.01050 |
| Zfp407 |  |  |  | 8.496 | 0.00957 |
| Cdhr3 |  |  |  | 8.408 | 0.02932 |
| LOC100048513 |  |  |  | 8.000 | 0.04677 |
| Lrrc14b |  |  |  | 7.035 | 0.04063 |
| Dbh |  |  |  | 6.355 | 0.03985 |
| Pdlim5 |  |  |  | 6.314 | 0.01225 |
| Slc25a18 |  |  |  | 6.291 | 0.02880 |
| LOC100047320 |  |  |  | 6.147 | 0.03107 |
| Gadl1 |  |  |  | 6.107 | 0.02675 |
| Gckr |  |  |  | 6.050 | 0.03042 |
| LOC669787 |  |  |  | 5.825 | 0.00709 |
| Smtnl1 |  |  |  | 5.458 | 0.01388 |
| 4933408N05Rik |  |  |  | 5.111 | 0.03556 |
| A2bp1 |  |  |  | 5.093 | 0.02044 |
| Cldn3 |  |  |  | 5.040 | 0.01050 |
| Myh4 | * |  |  | 4.639 | 0.02445 |
| Asph |  |  |  | 4.464 | 0.00709 |
| Ms4a10 |  |  |  | 4.370 | 0.02212 |
| Gm5091 |  |  |  | 4.180 | 0.02110 |
| Cela3b |  |  |  | 3.977 | 0.03055 |
| Tpm1 |  |  |  | 3.850 | 0.00371 |
| Serpina11 |  |  |  | 3.780 | 0.04081 |
| Gm8617 |  |  |  | 3.770 | 0.04568 |
| AA060545 |  |  |  | 3.592 | 0.04677 |
| Erbb4 |  |  |  | 3.370 | 0.00371 |
| Kcnj12 |  |  |  | 3.220 | 0.03387 |
| Prss46 |  |  |  | 3.155 | 0.02675 |
| Csn3 |  |  |  | 3.101 | 0.03075 |
| Palm2 |  |  |  | 2.939 | 0.00399 |
| Sept4 |  |  |  | 2.930 | 0.02751 |
| 4833419O12Rik |  |  |  | 2.909 | 0.04676 |
| 4930429F24Rik |  |  |  | 2.886 | 0.03856 |
| 4930511E03Rik |  |  |  | 2.863 | 0.01289 |
| Sirt7 |  |  |  | 2.862 | 0.03084 |
| Fam169b |  |  |  | 2.827 | 0.01050 |
| 6720420G18Rik |  |  |  | 2.715 | 0.04890 |
| 1700125H20Rik |  |  |  | 2.560 | 0.04677 |
| Atp2a2 |  |  |  | 2.485 | 0.04550 |
| 4833415N18Rik |  |  |  | 2.478 | 0.04328 |
| LOC634496 |  |  |  | 2.397 | 0.04176 |
| Has1 |  |  |  | 2.364 | 0.03442 |
| Rhox5 |  |  |  | 2.361 | 0.02906 |
| Speg |  |  |  | 2.311 | 0.04918 |
| Parm1 |  |  |  | 2.293 | 0.02212 |
| C230098O21Rik |  |  |  | 2.265 | 0.04554 |
| AI314760 |  |  |  | 2.195 | 0.04843 |
| Dusp8 |  |  |  | 2.145 | 0.02166 |
| Dpysl3 |  |  |  | 2.139 | 0.04554 |
| Celf1 |  |  |  | 2.127 | 0.02683 |
| Ehd4 |  |  |  | 2.093 | 0.02629 |
| Tmem100 |  |  |  | 2.089 | 0.01050 |
| 4922502B01Rik |  |  |  | 2.057 | 0.00709 |
| Abca8b |  |  |  | 2.009 | 0.02974 |

Supplementary Date Table 2: List of all DE up-regulated genes and fold change estimated by Microarray and RNA-seq.

| **Gene id** | **RNA sequencing**  **Up-regulated** | | **Microarray**  **Up-regulated** | |
| --- | --- | --- | --- | --- |
|  | Fold Change | p-value | Fold Change | p-value |
| Cntnap5c | Inf^[[2]](#footnote-2)^ | 0.00811168 |  |  |
| Htr4 | Inf | 0.015985671 |  |  |
| Phox2b | 40.652 | 0.021683372 |  |  |
| Oprk1 | 33.523 | 0.009989198 |  |  |
| Th | 14.885 | 0.019367024 |  |  |
| Olfm3 | 14.499 | 1.96471E-05 |  |  |
| Tinag | 13.962 | 0.006855256 |  |  |
| Aqp5 | 11.241 | 0.03827926 | 2.976 | 0.02829061 |
| Cntnap5b | 10.734 | 0.007798118 |  |  |
| Lrp1b | 10.356 | 2.67313E-10 |  |  |
| Stmn3 | 10.052 | 0.006003718 |  |  |
| Fstl5 | 9.993 | 0.031568933 |  |  |
| Pcp4 | 9.409 | 2.85327E-11 |  |  |
| Ces2g | 9.142 | 0.000183355 |  |  |
| Bhlhe22 | 9.129 | 0.006008402 |  |  |
| Kcnip1 | 8.704 | 8.61502E-09 | 5.874 | 0.026830187 |
| Ifi44 | 8.444 | 0.012913212 |  |  |
| Fam163a | 8.348 | 5.13568E-05 |  |  |
| Galntl6 | 8.086 | 0.000220113 |  |  |
| 3110047P20Rik | 7.627 | 0.038093175 |  |  |
| Tmem28 | 7.574 | 0.000653005 | 3.785 | 0.03042167 |
| Kcnj5 | 7.232 | 0.002870959 | 2.083 | 0.047536302 |
| Tmem213 | 7.231 | 0.000548183 |  |  |
| Nefh | 7.189 | 0.042118677 |  |  |
| Gm765 | 6.906 | 0.001092695 |  |  |
| Qrfpr | 6.904 | 0.018242304 |  |  |
| 9130404H23Rik | 6.805 | 0.013520604 |  |  |
| Itgb2l | 6.688 | 0.031025179 |  |  |
| Slc16a14 | 6.352 | 0.008701364 |  |  |
| 6330407J23Rik | 6.345 | 0.044510005 |  |  |
| Zpld1 | 6.175 | 0.010396425 |  |  |
| 3110082D06Rik | 6.083 | 3.90165E-06 |  |  |
| Grin2c | 6.058 | 0.012818752 |  |  |
| Frem3 | 5.962 | 5.38805E-06 |  |  |
| Adh7 | 5.875 | 0.01468373 |  |  |
| Spock1 | 5.682 | 5.8183E-15 |  |  |
| Grm8 | 5.669 | 9.60601E-07 |  |  |
| Lingo2 | 5.580 | 3.48887E-11 | 4.696 | 0.009014199 |
| Slfn10-ps | 5.435 | 0.033647487 |  |  |
| Rmst | 5.391 | 0.000311655 |  |  |
| Chrm2 | 5.227 | 0.001028715 |  |  |
| Kcnh3 | 5.208 | 0.000149503 |  |  |
| Lhcgr | 5.169 | 0.006109941 |  |  |
| Zfp804a | 5.100 | 6.23689E-06 |  |  |
| Nxph1 | 5.074 | 5.74355E-05 |  |  |
| Ina | 5.034 | 0.018844215 |  |  |
| Kcnh5 | 4.935 | 9.19928E-05 |  |  |
| Cer1 | 4.931 | 0.004938087 |  |  |
| Trhde | 4.916 | 3.38578E-13 |  |  |
| Lrrtm1 | 4.800 | 3.26074E-11 | 2.748 | 0.003270305 |
| Odz1 | 4.769 | 9.31145E-13 |  |  |
| Rasgrf2 | 4.726 | 6.94161E-09 |  |  |
| Hs3st5 | 4.725 | 0.018399541 |  |  |
| Gpr50 | 4.718 | 0.005342074 |  |  |
| Galnt13 | 4.712 | 2.28464E-05 |  |  |
| Irs4 | 4.699 | 8.89623E-05 |  |  |
| Gabrg3 | 4.615 | 0.014084194 |  |  |
| Pde11a | 4.554 | 4.57853E-06 |  |  |
| Camp | 4.524 | 5.80342E-05 |  |  |
| Cd96 | 4.494 | 2.02484E-05 | 6.327 | 0.007088687 |
| Emr4 | 4.421 | 0.048807742 |  |  |
| Cdh8 | 4.394 | 2.04332E-11 |  |  |
| Darc | 4.384 | 0.011011717 | 2.360 | 0.046771158 |
| Kcnc2 | 4.369 | 0.002316834 |  |  |
| Grem2 | 4.358 | 6.87843E-18 | 4.909 | 0.0191443 |
| Sstr4 | 4.334 | 0.000478644 |  |  |
| Kctd16 | 4.312 | 0.003200397 |  |  |
| Zfp804b | 4.251 | 0.009898747 |  |  |
| Gpr88 | 4.242 | 2.11632E-09 | 3.497 | 0.020233307 |
| Gabra5 | 4.198 | 3.91689E-05 |  |  |
| Cdh20 | 4.194 | 5.57411E-09 |  |  |
| Ltf | 4.172 | 0.00010046 |  |  |
| Odz2 | 4.065 | 1.64435E-16 |  |  |
| Mdga2 | 4.019 | 3.19678E-10 |  |  |
| Rassf6 | 4.013 | 0.000941612 |  |  |
| Cldn11 | 4.012 | 5.82596E-19 | 4.339 | 0.026749311 |
| Olfm4 | 3.995 | 0.000368956 |  |  |
| Pappa | 3.983 | 2.52103E-18 |  |  |
| Cxcl5 | 3.977 | 0.007045612 |  |  |
| Nebl | 3.977 | 3.43655E-09 |  |  |
| Wnt2 | 3.958 | 0.000104042 |  |  |
| Mx2 | 3.922 | 0.011710449 |  |  |
| Samd12 | 3.863 | 0.030721288 |  |  |
| Kcna2 | 3.848 | 0.000649711 |  |  |
| Dscam | 3.841 | 0.000206703 |  |  |
| BC100530 | 3.818 | 0.035898844 |  |  |
| Sorcs3 | 3.813 | 7.88158E-09 | 3.265 | 0.024911175 |
| Chd5 | 3.809 | 2.06004E-05 | 2.339 | 0.02829061 |
| Dnahc2 | 3.805 | 1.6188E-10 |  |  |
| Cyp11a1 | 3.743 | 0.00526559 |  |  |
| Pcdh8 | 3.734 | 1.37966E-10 | 5.244 | 0.006449103 |
| Pcdh11x | 3.718 | 4.83242E-09 |  |  |
| Adora3 | 3.680 | 0.021555195 |  |  |
| St6gal2 | 3.627 | 0.000126933 |  |  |
| Nrxn3 | 3.625 | 0.001157601 | 2.646 | 0.046771158 |
| Lamc3 | 3.587 | 0.003367514 |  |  |
| Lrrc7 | 3.584 | 0.000132273 |  |  |
| Syt2 | 3.563 | 0.014669478 |  |  |
| S100a9 | 3.546 | 2.28122E-09 |  |  |
| Wnt2b | 3.497 | 9.69304E-09 |  |  |
| Sez6l | 3.469 | 3.89725E-07 |  |  |
| Rtp4 | 3.467 | 1.40937E-05 |  |  |
| Wfikkn2 | 3.466 | 3.77228E-20 | 3.457 | 0.007088687 |
| Apod | 3.439 | 9.15031E-10 | 2.863 | 0.04700062 |
| Ngp | 3.393 | 1.11712E-06 |  |  |
| Rag1 | 3.383 | 0.000298643 |  |  |
| Grap2 | 3.366 | 0.027143322 |  |  |
| Negr1 | 3.354 | 2.19112E-11 | 2.904 | 0.008664596 |
| Slitrk3 | 3.351 | 2.77517E-07 |  |  |
| Pnma2 | 3.348 | 0.000388047 |  |  |
| Bnc2 | 3.326 | 5.17221E-08 |  |  |
| Ankrd55 | 3.305 | 0.000166176 |  |  |
| Lrrn4 | 3.294 | 0.007133272 |  |  |
| Tmem90b | 3.271 | 0.001382144 |  |  |
| Tulp2 | 3.263 | 0.007094287 |  |  |
| Plxna4 | 3.258 | 1.04676E-05 | 2.648 | 0.03042167 |
| Nrg1 | 3.251 | 1.13062E-05 | 2.866 | 0.029743126 |
| Glis3 | 3.250 | 8.99409E-05 |  |  |
| 2010110P09Rik | 3.230 | 0.034177208 |  |  |
| Lypd1 | 3.229 | 0.012599147 |  |  |
| Erc2 | 3.226 | 3.41887E-11 | 2.630 | 0.022361835 |
| Lgals7 | 3.182 | 0.004911859 |  |  |
| Lrrn3 | 3.178 | 1.13647E-10 | 3.170 | 0.012865157 |
| Figf | 3.173 | 1.54196E-06 | 3.515 | 0.03106743 |
| Cadm3 | 3.151 | 6.5051E-09 |  |  |
| Cd274 | 3.146 | 0.044689098 |  |  |
| Lama3 | 3.138 | 1.65109E-11 |  |  |
| Gprin3 | 3.130 | 0.013194692 |  |  |
| Lhfpl3 | 3.105 | 0.004501974 |  |  |
| Grin2a | 3.105 | 0.001212279 |  |  |
| Il2rb | 3.093 | 0.030047996 |  |  |
| Vstm2a | 3.090 | 1.06252E-06 | 8.556 | 0.022071414 |
| Grin3a | 3.083 | 6.59045E-06 |  |  |
| Npy2r | 3.076 | 0.043465598 | 4.083 | 0.016104808 |
| Wnt16 | 3.065 | 0.000678927 |  |  |
| Aff2 | 3.061 | 7.0861E-07 |  |  |
| Bdh2 | 3.057 | 2.14413E-07 | 3.045 | 0.046683427 |
| Chrm1 | 3.047 | 0.009294854 | 5.696 | 0.0432783 |
| Chgb | 2.987 | 0.015373266 |  |  |
| Cpa3 | 2.961 | 2.02486E-08 |  |  |
| Fat4 | 2.957 | 2.48032E-14 |  |  |
| Nhs | 2.954 | 7.42082E-07 |  |  |
| Dsc3 | 2.944 | 7.23407E-07 |  |  |
| Prtn3 | 2.943 | 0.01258186 |  |  |
| Gm13154 | 2.930 | 0.029073583 |  |  |
| Lrfn2 | 2.926 | 1.53587E-06 | 2.999 | 0.04700292 |
| Mme | 2.916 | 4.60047E-10 |  |  |
| Stfa1 | 2.915 | 0.000355257 |  |  |
| Slc26a7 | 2.907 | 7.61292E-06 |  |  |
| Gsg1l | 2.892 | 4.18696E-10 |  |  |
| C030030A07Rik | 2.879 | 0.013809116 |  |  |
| Grpr | 2.872 | 0.000323907 |  |  |
| Kcnd2 | 2.853 | 1.07959E-11 |  |  |
| Sned1 | 2.847 | 0.000172413 |  |  |
| Unc13c | 2.835 | 0.000352109 |  |  |
| Rasgrf1 | 2.834 | 0.025886378 |  |  |
| Esr1 | 2.834 | 0.042051048 |  |  |
| Hgf | 2.833 | 1.62534E-06 |  |  |
| Cacna2d3 | 2.822 | 3.46203E-08 | 2.877 | 0.03042167 |
| Pcdhb2 | 2.811 | 0.004118991 |  |  |
| Trim30d | 2.806 | 0.032401337 |  |  |
| Kctd8 | 2.802 | 0.014080311 |  |  |
| Zfp366 | 2.798 | 0.014737195 |  |  |
| D830031N03Rik | 2.790 | 0.023177946 |  |  |
| Rasd2 | 2.786 | 0.007335242 |  |  |
| Slc22a3 | 2.780 | 0.00226207 |  |  |
| Pgr | 2.773 | 0.017805759 |  |  |
| Aox3 | 2.764 | 0.017855151 |  |  |
| Cldn1 | 2.734 | 1.78588E-11 |  |  |
| Gprin2 | 2.732 | 0.014645987 |  |  |
| Mgat4c | 2.727 | 0.003141679 |  |  |
| Csmd3 | 2.720 | 8.12645E-08 |  |  |
| Rassf9 | 2.718 | 0.001300208 |  |  |
| Ifltd1 | 2.707 | 0.021396163 |  |  |
| Pi15 | 2.702 | 2.92394E-07 |  |  |
| Aldh1a1 | 2.702 | 0.031247555 |  |  |
| Ptchd1 | 2.700 | 1.32068E-06 |  |  |
| Rims1 | 2.696 | 0.00361253 |  |  |
| Lmx1a | 2.693 | 0.002502881 |  |  |
| Vnn1 | 2.685 | 0.001229868 |  |  |
| Tpsg1 | 2.683 | 0.013827512 |  |  |
| S100a8 | 2.682 | 0.001179789 |  |  |
| Aox4 | 2.680 | 0.005330953 |  |  |
| Gbp3 | 2.680 | 0.020948944 |  |  |
| Cadm2 | 2.669 | 5.97444E-05 |  |  |
| Thsd7b | 2.669 | 3.65584E-08 |  |  |
| Stac2 | 2.664 | 1.06734E-05 | 2.518 | 0.021104623 |
| Uprt | 2.660 | 0.036481628 |  |  |
| Pcdhb6 | 2.657 | 0.000971057 |  |  |
| Syt5 | 2.657 | 0.014578781 |  |  |
| 3110099E03Rik | 2.652 | 0.028989904 |  |  |
| Clic5 | 2.644 | 0.000143118 | 2.598 | 0.015984364 |
| Robo2 | 2.638 | 4.77656E-13 |  |  |
| Optc | 2.636 | 0.006670803 |  |  |
| Bai3 | 2.634 | 9.03636E-07 |  |  |
| Gria2 | 2.630 | 0.003821262 |  |  |
| Hdc | 2.627 | 0.00789997 |  |  |
| Edar | 2.626 | 0.034376024 |  |  |
| A730069N07Rik | 2.624 | 0.037926155 |  |  |
| Rtn4r | 2.620 | 3.29944E-05 |  |  |
| Oprd1 | 2.615 | 0.015080163 |  |  |
| Mmp28 | 2.608 | 0.004942454 |  |  |
| Gpc5 | 2.607 | 0.001010127 |  |  |
| Dbc1 | 2.607 | 6.89248E-07 |  |  |
| Flrt1 | 2.604 | 4.38868E-06 |  |  |
| H60b | 2.593 | 0.028096446 |  |  |
| Oprl1 | 2.566 | 0.006032349 |  |  |
| Fmn2 | 2.564 | 7.04459E-06 |  |  |
| Pgap1 | 2.548 | 9.99216E-05 |  |  |
| 9130008F23Rik | 2.537 | 0.016995569 |  |  |
| Hbb-bh1 | 2.533 | 0.000587881 |  |  |
| Grid2 | 2.533 | 0.004232619 |  |  |
| Pcdh10 | 2.529 | 9.72004E-12 | 2.565 | 0.017431902 |
| Arhgap20 | 2.516 | 1.02353E-10 |  |  |
| Adamts12 | 2.514 | 6.89168E-05 |  |  |
| Grm4 | 2.514 | 0.000987148 |  |  |
| Siglec1 | 2.511 | 0.013051847 |  |  |
| Scg2 | 2.507 | 0.019336708 |  |  |
| Mmrn1 | 2.496 | 5.54907E-07 |  |  |
| Kcna1 | 2.487 | 6.31412E-09 |  |  |
| Scube2 | 2.486 | 1.19351E-08 |  |  |
| Lmx1b | 2.479 | 3.20234E-05 |  |  |
| Lrrc4c | 2.475 | 4.20325E-07 |  |  |
| Gm106 | 2.474 | 5.60136E-06 |  |  |
| Sorcs1 | 2.471 | 9.0741E-11 |  |  |
| Tcfcp2l1 | 2.467 | 0.00035783 |  |  |
| Thsd4 | 2.457 | 4.74163E-11 |  |  |
| Trpc5 | 2.450 | 0.044192008 |  |  |
| Dlg2 | 2.449 | 5.29662E-09 |  |  |
| Cntn4 | 2.441 | 0.00027323 |  |  |
| Klhl34 | 2.438 | 0.007011755 |  |  |
| Dkk2 | 2.436 | 1.28371E-08 |  |  |
| Fat2 | 2.433 | 0.000122468 |  |  |
| Rspo2 | 2.428 | 2.24518E-10 | 3.077 | 0.011657668 |
| Sesn3 | 2.423 | 1.31833E-05 |  |  |
| Sorl1 | 2.422 | 3.60514E-07 | 2.193 | 0.03170701 |
| H2-T24 | 2.407 | 0.011510566 |  |  |
| Vsnl1 | 2.407 | 0.001124722 |  |  |
| Tmem26 | 2.405 | 4.68593E-11 |  |  |
| Gabrb3 | 2.400 | 8.28704E-08 |  |  |
| Rgs9bp | 2.394 | 0.000213765 |  |  |
| Crabp1 | 2.392 | 6.36088E-07 | 2.820 | 0.022896456 |
| Fgf2 | 2.389 | 0.006861295 |  |  |
| Gpr21 | 2.388 | 0.049713237 |  |  |
| Ahr | 2.385 | 1.25812E-08 |  |  |
| Lsamp | 2.382 | 0.000115077 |  |  |
| Lcn2 | 2.380 | 0.00536202 |  |  |
| Zfp369 | 2.379 | 4.66917E-05 |  |  |
| Slit3 | 2.378 | 3.61817E-05 |  |  |
| Pcdhb10 | 2.375 | 0.014264068 |  |  |
| Tuba8 | 2.374 | 0.045281056 |  |  |
| Hcn1 | 2.374 | 0.000443861 |  |  |
| Accn1 | 2.371 | 0.030431602 |  |  |
| Maf | 2.370 | 8.046E-11 |  |  |
| Gstt1 | 2.369 | 9.34437E-06 | 2.966 | 0.014203158 |
| Epha4 | 2.367 | 1.09558E-06 |  |  |
| Cadps2 | 2.367 | 2.02583E-07 |  |  |
| Foxc2 | 2.362 | 3.77885E-09 | 2.091 | 0.022120591 |
| Samd9l | 2.359 | 4.06757E-05 |  |  |
| Pde3a | 2.356 | 0.000252521 |  |  |
| Cnksr2 | 2.350 | 1.67466E-06 | 2.034 | 0.023361117 |
| Olfml2a | 2.348 | 0.004423579 |  |  |
| Rundc2a | 2.339 | 0.000568975 |  |  |
| Prdm11 | 2.339 | 0.007433865 |  |  |
| Cxcl13 | 2.334 | 0.000815449 |  |  |
| 2410066E13Rik | 2.333 | 5.12866E-05 |  |  |
| Ptx3 | 2.327 | 4.83727E-10 | 2.724 | 0.009014199 |
| Ptafr | 2.325 | 0.012971943 |  |  |
| Mecom | 2.324 | 9.89019E-10 |  |  |
| Pcdhga2 | 2.322 | 4.60663E-05 |  |  |
| Prrx1 | 2.321 | 9.53569E-11 |  |  |
| Foxd2 | 2.318 | 2.39873E-06 |  |  |
| Xpnpep2 | 2.318 | 1.89682E-05 |  |  |
| Adra1d | 2.314 | 0.021633553 |  |  |
| Cbl | 2.307 | 0.010130391 |  |  |
| Plb1 | 2.306 | 0.028733823 |  |  |
| Adra1b | 2.304 | 0.003433491 |  |  |
| Enpep | 2.299 | 2.53756E-06 |  |  |
| Ccdc158 | 2.295 | 0.045877342 |  |  |
| Ncam2 | 2.295 | 0.002124326 |  |  |
| Col8a1 | 2.295 | 1.09688E-09 |  |  |
| Myocd | 2.292 | 0.040200711 |  |  |
| Ankrd45 | 2.288 | 0.047353982 |  |  |
| Slc40a1 | 2.286 | 1.12932E-09 | 2.326 | 0.006270704 |
| Angptl4 | 2.282 | 0.004535418 | 2.277 | 0.030546911 |
| Abca1 | 2.280 | 1.51947E-09 |  |  |
| Pdpr | 2.277 | 0.007641978 |  |  |
| Cntn1 | 2.275 | 9.40642E-07 | 2.286 | 0.029743126 |
| Slitrk6 | 2.274 | 3.4985E-08 |  |  |
| Zfp382 | 2.273 | 0.025343336 |  |  |
| Epha5 | 2.273 | 8.58459E-08 |  |  |
| Mgat5 | 2.272 | 0.027815516 |  |  |
| Hhip | 2.265 | 5.90086E-05 |  |  |
| 4930441O14Rik | 2.265 | 0.034203402 |  |  |
| Sox5 | 2.263 | 0.018114271 |  |  |
| Kif13b | 2.260 | 0.001190356 |  |  |
| Svep1 | 2.258 | 1.16294E-05 |  |  |
| Dnahc8 | 2.256 | 0.001986264 |  |  |
| Dlx6os1 | 2.255 | 0.000714783 | 2.995 | 0.022896456 |
| Prdm6 | 2.255 | 1.31907E-06 | 2.316 | 0.020373477 |
| Syt1 | 2.252 | 0.028025215 |  |  |
| Hyal1 | 2.249 | 0.000200445 |  |  |
| Dio3 | 2.249 | 0.003613222 |  |  |
| Shroom4 | 2.249 | 0.002278556 |  |  |
| Samd5 | 2.248 | 0.020010474 |  |  |
| Fam198a | 2.247 | 9.27339E-09 |  |  |
| Sema5a | 2.246 | 1.05617E-09 |  |  |
| Hbb-y | 2.243 | 0.038467745 |  |  |
| Krt7 | 2.243 | 0.000961023 |  |  |
| Zbed6 | 2.241 | 0.019980525 |  |  |
| Xrn1 | 2.231 | 0.006731401 |  |  |
| Fam84a | 2.228 | 2.96287E-07 |  |  |
| Rassf8 | 2.223 | 0.000601203 |  |  |
| Fam107a | 2.223 | 0.004755457 | 2.136 | 0.022120591 |
| Fgd3 | 2.223 | 0.030165821 |  |  |
| Prokr1 | 2.222 | 3.35816E-08 |  |  |
| Hmbox1 | 2.219 | 0.003467061 |  |  |
| Serpinb1a | 2.218 | 0.000184334 |  |  |
| Ppp1r3b | 2.212 | 1.4059E-08 |  |  |
| Ldb2 | 2.212 | 2.9089E-09 |  |  |
| Bace2 | 2.211 | 6.73151E-06 | 2.835 | 0.030364744 |
| Elovl7 | 2.200 | 0.007187013 |  |  |
| Kcns3 | 2.199 | 2.78753E-06 |  |  |
| Stk32b | 2.198 | 9.71846E-07 |  |  |
| Zkscan16 | 2.197 | 0.002988075 |  |  |
| Cntn5 | 2.196 | 0.013216323 |  |  |
| Gan | 2.192 | 0.00130235 |  |  |
| Fmo1 | 2.191 | 0.001376719 |  |  |
| Krt15 | 2.190 | 0.024841916 | 5.601 | 0.036359612 |
| Kcnb2 | 2.190 | 0.041471176 | 2.901 | 0.041941773 |
| Foxo3 | 2.189 | 0.000246334 |  |  |
| L3mbtl3 | 2.189 | 6.73263E-09 |  |  |
| Lgr5 | 2.187 | 6.19424E-07 |  |  |
| Col8a2 | 2.187 | 8.85889E-10 |  |  |
| Rgs7bp | 2.183 | 4.93168E-06 |  |  |
| Tox | 2.182 | 4.55433E-08 |  |  |
| Thsd7a | 2.179 | 7.40566E-06 |  |  |
| Pcdhga1 | 2.175 | 0.000730494 |  |  |
| BC005561 | 2.175 | 0.006359051 |  |  |
| Gfra2 | 2.173 | 3.02507E-08 |  |  |
| Cd83 | 2.173 | 1.31811E-05 | 2.157 | 0.0432783 |
| Dpyd | 2.171 | 3.1538E-06 | 2.310 | 0.02829061 |
| Pappa2 | 2.170 | 0.000174666 |  |  |
| Alcam | 2.165 | 9.22825E-10 |  |  |
| Scube3 | 2.161 | 0.000109247 |  |  |
| Cxcl14 | 2.161 | 2.3385E-06 |  |  |
| Ccbe1 | 2.159 | 4.98708E-07 |  |  |
| Rapgef5 | 2.159 | 2.88791E-05 |  |  |
| Masp1 | 2.158 | 0.006495732 |  |  |
| Cdkl5 | 2.155 | 0.026246787 |  |  |
| 6430704M03Rik | 2.150 | 0.003661461 |  |  |
| Kcnab1 | 2.146 | 3.71027E-05 |  |  |
| Nckap5 | 2.145 | 0.000387554 |  |  |
| Ace | 2.144 | 0.003928341 |  |  |
| Spock2 | 2.144 | 1.40746E-07 |  |  |
| Ptch1 | 2.143 | 0.014230432 |  |  |
| Klf7 | 2.143 | 0.008730025 |  |  |
| N28178 | 2.142 | 0.03120798 |  |  |
| Trim30a | 2.142 | 0.000562132 |  |  |
| Paqr6 | 2.142 | 0.010785309 |  |  |
| Opcml | 2.140 | 1.0228E-05 |  |  |
| Sema3d | 2.140 | 0.000504096 |  |  |
| Fgf10 | 2.139 | 8.88607E-08 | 2.478 | 0.03668818 |
| Tns3 | 2.139 | 8.54283E-09 |  |  |
| Tlr7 | 2.137 | 0.003542479 |  |  |
| Gucy1a2 | 2.135 | 0.028616299 |  |  |
| Gbp6 | 2.131 | 0.000161545 |  |  |
| Apbb2 | 2.130 | 0.000665099 |  |  |
| Rnf182 | 2.128 | 0.000280459 |  |  |
| Igf1 | 2.124 | 5.59649E-09 |  |  |
| Cbln1 | 2.123 | 0.014485088 |  |  |
| Rassf4 | 2.118 | 7.75766E-08 |  |  |
| Hmcn1 | 2.117 | 1.17611E-05 |  |  |
| Pcdhga9 | 2.115 | 0.001917222 |  |  |
| Spn | 2.109 | 0.038737032 |  |  |
| Elavl2 | 2.108 | 0.003871433 |  |  |
| C3 | 2.106 | 0.001376546 |  |  |
| Grm7 | 2.106 | 4.09248E-06 |  |  |
| Cpz | 2.104 | 0.014086119 |  |  |
| Scn1a | 2.102 | 0.01030945 |  |  |
| Lrfn5 | 2.100 | 0.007871477 |  |  |
| Epha3 | 2.097 | 5.43178E-07 |  |  |
| Sfrp2 | 2.092 | 8.71221E-09 | 2.618 | 0.013549614 |
| Chrna7 | 2.091 | 0.00528383 |  |  |
| Enam | 2.088 | 0.03526418 |  |  |
| Sarm1 | 2.086 | 0.003770446 |  |  |
| Evi2a | 2.085 | 0.007561705 |  |  |
| Rnf180 | 2.079 | 0.008231714 |  |  |
| Ptplad2 | 2.077 | 0.002338624 |  |  |
| Krt18 | 2.075 | 1.68256E-05 | 2.290 | 0.024764035 |
| Il1rl1 | 2.067 | 0.000501689 |  |  |
| Zfp618 | 2.064 | 0.000605746 |  |  |
| Hba-x | 2.064 | 4.27266E-05 |  |  |
| Megf9 | 2.063 | 0.005656898 |  |  |
| Trim56 | 2.062 | 0.023901364 |  |  |
| Slc16a8 | 2.062 | 0.02993116 |  |  |
| Grp | 2.059 | 0.016656605 |  |  |
| Ccdc30 | 2.057 | 0.015181653 |  |  |
| Slc4a11 | 2.048 | 0.016905863 |  |  |
| Hmgcs2 | 2.046 | 0.009096192 |  |  |
| Mpo | 2.042 | 0.026482552 |  |  |
| Pdgfra | 2.041 | 8.36077E-08 |  |  |
| Shank1 | 2.039 | 0.019115693 |  |  |
| Klf12 | 2.034 | 4.99436E-05 |  |  |
| Bmp3 | 2.030 | 3.60153E-06 |  |  |
| Wnt4 | 2.028 | 0.000376716 |  |  |
| Gm13152 | 2.027 | 0.002657245 |  |  |
| Rspo3 | 2.022 | 9.07763E-08 |  |  |
| Lonrf2 | 2.020 | 7.45703E-06 |  |  |
| Kitl | 2.017 | 2.23348E-07 |  |  |
| 1700003E16Rik | 2.017 | 0.000337161 |  |  |
| Megf6 | 2.016 | 0.00033747 | 2.069 | 0.019919738 |
| Zbtb7c | 2.011 | 1.04392E-05 |  |  |
| Lphn3 | 2.011 | 6.2356E-05 |  |  |
| D130017N08Rik | 2.009 | 0.012273568 |  |  |
| Ccdc60 | 2.007 | 0.001639865 |  |  |
| Rab17 | 2.006 | 0.030860461 |  |  |
| Slc5a3 | 2.004 | 4.00678E-07 |  |  |
| Syt16 | 2.001 | 0.000316039 |  |  |
| Kndc1 |  |  | 5.317 | 0.013908912 |
| LOC100046616 |  |  | 4.014 | 0.04956006 |
| Ccdc121 |  |  | 3.313 | 0.03042167 |
| Gm5382 |  |  | 3.244 | 0.024911175 |
| 1700025F22Rik |  |  | 3.023 | 0.015984364 |
| Stat1 |  |  | 2.952 | 0.030546911 |
| Slc5a7 |  |  | 2.908 | 0.021124505 |
| Gpr4 |  |  | 2.836 | 0.04425495 |
| AA387883 |  |  | 2.759 | 0.008290993 |
| Gm3333 |  |  | 2.724 | 0.030546911 |
| Nrip3 |  |  | 2.627 | 0.030546911 |
| Nrxn1 |  |  | 2.587 | 0.03586748 |
| Tmem178 |  |  | 2.561 | 0.006270704 |
| Syn2 |  |  | 2.460 | 0.020233307 |
| Penk |  |  | 2.453 | 0.024911175 |
| Krt13 |  |  | 2.427 | 0.04227364 |
| E330013P04Rik |  |  | 2.410 | 0.03668818 |
| Fxyd7 |  |  | 2.325 | 0.024911175 |
| Mgst1 |  |  | 2.283 | 0.035782292 |
| LOC552880 |  |  | 2.278 | 0.034443438 |
| Tmem145 |  |  | 2.270 | 0.012821585 |
| 5033411D12Rik |  |  | 2.227 | 0.04737632 |
| Rnd1 |  |  | 2.213 | 0.047857255 |
| Me3 |  |  | 2.207 | 0.026873125 |
| Stmn2 |  |  | 2.190 | 0.0417661 |
| Fam196a |  |  | 2.181 | 0.012889229 |
| Fam181b |  |  | 2.159 | 0.005028295 |
| Gal3st4 |  |  | 2.119 | 0.029877413 |
| Sfrs13b |  |  | 2.106 | 0.010502099 |
| Sult5a1 |  |  | 2.088 | 0.046443906 |
| 1500015O10Rik |  |  | 2.050 | 0.027150191 |
| BC068157 |  |  | 2.016 | 0.048310697 |
| Ankrd29 |  |  | 2.005 | 0.033869874 |

| Enrichment of muscle associated genes identified in the down-regulated gene category | | | |
| --- | --- | --- | --- |
| **Gene Ontology Term** | | **Gene count in study** | **Enrichment^1^** |
| **Muscle Development/ Structure/ Function** | | [69] | 1.77 x10^-25^ to 2.42 x10^-8^ |
|  | GO:0007517: muscle organ development | 43 | 1.77 x10^-25^ |
|  | GO:0060537: muscle tissue development | 35 | 1.58 x10^-21^ |
|  | GO:0014706: striated muscle tissue development | 34 | 1.69 x10^-21^ |
|  | GO:0055002: striated muscle cell development | 23 | 1.63 x10^-19^ |
|  | GO:0051146: striated muscle cell differentiation | 27 | 1.36 x10^-18^ |
|  | GO:0003012: muscle system process | 24 | 2.32 x10^-18^ |
|  | GO:0006936: muscle contraction | 23 | 2.47 x10^-18^ |
|  | GO:0055001: muscle cell development | 23 | 3.74 x10^-18^ |
|  | GO:0042692: muscle cell differentiation | 29 | 2.11 x10^-17^ |
|  | GO:0007519: skeletal muscle tissue development | 22 | 3.22 x10^-15^ |
|  | GO:0060538: skeletal muscle organ development | 22 | 5.97 x10^-15^ |
|  | GO:0006941: striated muscle contraction | 10 | 2.42 x10^-8^ |
| 1. P-value of enrichment of GO terms using DAVID analysis software; similar results were found with GOstat. | | | |

Supplementary Data Table 4:

1. Fold Change not determined as no expression in Mutant [↑](#footnote-ref-1)
2. Fold Change not determined as no expression in Control [↑](#footnote-ref-2)
